# Supplementary material for: Melatonin agonist tasimelteon (HETLIOZ®) improves sleep in patients with primary insomnia: A multicenter, randomized, double-blind, placebo-controlled trial
Source: PLoS One. 2025 Sep 19;20(9):e0332366. doi: 10.1371/journal.pone.0332366 (PMC12449008; doi:10.1371/journal.pone.0332366)
Supplement: S1 Protocol — (PDF) [file pone.0332366.s001.pdf]

VEC-162

VP-VEC-162-3104 AMENDMENT NO. 1

A MULTICENTER, RANDOMIZED, DOUBLE-BLIND, PLACEBO-CONTROLLED, PARALLEL STUDY TO INVESTIGATE THE EFFICACY AND SAFETY OF VEC-162 (20 MG/DAY AND 50 MG/DAY) IN THE TREATMENT OF PRIMARY INSOMNIA.

**Authors:**	Rosarelis Torres, Ph.D. and Christin Scott, M.S.
**Document Type:**	Clinical Study Protocol
**Sponsor:**	Vanda Pharmaceuticals Inc.<br>9605 Medical Center Drive<br>Suite 300<br>Rockville, MD 20850
**Study Product:**	VEC-162
**Protocol Number:**	VP-VEC-162-3104
**Study Phase:**	III
**IND Number:**	54,776

Date: August 20, 2007
Status: Final
Number of Pages: 74

1. SYNOPSIS

**Name of Sponsor/Company:**<br>Vanda Pharmaceuticals Inc.
**Name of Investigational Product:**<br>VEC-162
**Name of Active Ingredient:**<br>VEC-162
**Title of Study:**<br>A multicenter, randomized, double-blind, placebo-controlled, parallel study to investigate the efficacy and safety of VEC-162 (20 mg/day and 50 mg/day) in the treatment of primary insomnia.
**Study center(s): Multicenter**
**Studied period:**<br>Estimated date first patient enrolled: Sept/Oct 2007<br>Estimated study duration: 9 months<br>**Phase of development:**<br>III
**Number of patients (planned):**<br>Approximately 324 eligible patients will be randomized and assigned to one of three treatment groups (VEC-162 20 mg, VEC-162 50 mg or placebo) in a 1:1:1 ratio. Treatment assignments will be made according to a randomization schedule.
**Diagnosis and main criteria for inclusion:**<br>Males or females 18-64 years of age, inclusive, with a diagnosis of primary insomnia based upon DSM-IV and PSG criteria.
**Investigational product, dosage and mode of administration:**<br>VEC-162 will be administered orally as doses of 20 mg or 50 mg. All capsules will be size 1 and the color will be opaque.
**Duration of treatment:**<br>Up to 47 days total: 7-days (± 2 days) of single-blind placebo lead-in, 35-days (± 2 days) of double-blind study treatment and 1-day of single-blind placebo wash-out period.
**Reference therapy, dosage and mode of administration:**<br>Placebo capsules will be provided in size and appearance identical to those containing VEC-162 and will be administered orally.
**Objectives:**<br>**Primary:**<br>1. To assess the effects of 20 and 50 mg VEC-162 on the average latency to persistent sleep (LPS) as measured by polysomnography (PSG) on Nights 1 and 8.<br>**Secondary:**<br>1. To assess the effects of 20 and 50 mg VEC-162 on the average Wake After Sleep Onset (WASO), total sleep time (TST), and sleep efficiency (SE) as measured by PSG on

Nights 1 and 8.

2. To assess the maintenance of effect of oral doses of 20 and 50 mg VEC-162 on the average LPS, WASO, TST, and SE as measured by PSG on Nights 22 and 29.
3. To assess the effects of 20 and 50 mg VEC-162 on PSG parameters for each individual evaluation night.
4. To assess the effects of single and multiple oral doses of 20 and 50 mg VEC-162 on subjective assessments such as sleep quality, depth of sleep, sleep latency, total sleep time, daytime function and daytime alertness.
5. To assess the next day residual effects of oral doses of 20 and 50 mg VEC-162 as measured by digit symbol substitution test (DSST) and visual analog scale (VAS).
6. To examine the potential rebound effects upon abrupt treatment cessation after 5 weeks of VEC-162 as measured by PSG.
7. To explore the safety and tolerability of multiple oral doses of 20 and 50 mg of VEC-162.
8. To explore subjective withdrawal symptoms after 5 weeks of VEC-162 treatment as measured by benzodiazepine withdrawal symptom questionnaire (BWSQ).
9. To explore the contribution of a polymorphism in the PER3 gene on the primary and secondary efficacy endpoints.

Overall Design:

This is a multicenter, randomized, double-blind, placebo-controlled, parallel study to investigate the efficacy and safety of VEC-162 and matching placebo in male and female patients with primary insomnia. The study will be divided into 2 phases: the pre-randomization phase and the randomization phase. The pre-randomization phase consists of the screening visit and a one week single-blind placebo lead-in that includes two consecutive nights of PSG assessments. The randomization phase consists of a 5 week double-blind evaluation period and a 1-night single-blind placebo wash-out period. During the double-blind evaluation period, patients will undergo four overnight visits [Nights 1, 8, 22 and 29 (± 2 days)] in which PSG will be conducted. Patients will return to the clinic on Night 36 for an additional night of PSG assessment with single-blind placebo treatment.

Criteria for evaluation:

Efficacy:

The primary efficacy parameter for this study will be LPS. The secondary efficacy variables will be WASO, TST, and SE as measured by polysomnography over an 8 hour opportunity to sleep. Additional secondary variables include subjective measures such as sleep quality, depth of sleep, sleep latency, total sleep time, daytime function and daytime alertness as measured by pre-sleep questionnaire (Pre-SQ) and post- sleep questionnaire (PSQ).

Safety:

The safety and tolerability measures will include the recording of adverse events (AEs), clinical laboratory evaluations, vital signs, and electrocardiograms (ECGs).

DSST and VAS will be used to assess next day residual effect.

BWSQ and PSG sleep parameters administered upon treatment cessation will be used to assess rebound insomnia and withdrawal symptoms.

Statistical methods:
If not otherwise specified, statistical significance is defined as $p \leq 0.05$ and is two-tailed.

The primary efficacy variable is the average LPS value from Nights 1 and 8. The primary efficacy variable will be analyzed using an ANCOVA model with treatment and (pooled) site as main effects, baseline as a covariate, and treatment-by-baseline interaction term (which will be removed from the model if not statistically significant at the 0.10 level). The primary efficacy analysis will be based on the Full Analysis set (modified ITT) data.

The statistical analyses will be detailed in the Statistical Analysis Plan.

2. TABLE OF CONTENTS AND LIST OF TABLES

TABLE OF CONTENTS

1. SYNOPSIS 3
2. TABLE OF CONTENTS AND LIST OF TABLES 6
3. LIST OF ABBREVIATIONS AND DEFINITIONS OF TERMS....11
4. INTRODUCTION 15
4.1. Background....15
4.2. Relevant Data Summary 16
4.3. Clinical Data 16
4.3.1. VP-VEC-162-2101 16
4.3.2. VP-VEC-162-3101 17
5. TRIAL OBJECTIVES AND RATIONALE 19
5.1. Objectives 19
5.1.1. Primary Objective....19
5.1.2. Secondary objectives 19
5.2. Rationale....19
5.2.1. Rationale for Dose and Schedule Selection....19
5.2.2. Rationale for Study Design....20
6. INVESTIGATIONAL PLAN....21
6.1. Overall Study Design and Plan: Description....21
6.1.1. Pre-randomization Phase 21
6.1.2. Randomization Phase....22
7. SELECTION AND WITHDRAWAL OF PATIENTS....25
7.1. Patient Inclusion Criteria 25
7.2. Patient Exclusion Criteria....26
7.3. Patient Withdrawal Criteria 27
8. TREATMENT OF PATIENTS 29
8.1. Dosing....29
8.2. Concomitant Medications....29
8.3. Treatment Compliance....30
8.4. Treatment Assignment and Randomization....30
8.4.1. Treatment Assignment....30
8.4.2. Randomization....31

9. STUDY DRUG MATERIALS AND MANAGEMENT 32
9.1. Study Drug 32
9.2. Study Drug Packaging and Labeling 32
9.3. Study Drug Storage 33
9.4. Study Drug Accountability 33
10. STUDY ASSESSMENTS AND RESTRICTIONS 35
10.1. Pre-Randomization 35
10.1.1. Screening Visit 35
10.1.2. Single-blind Placebo Lead-in Period 35
10.2. Randomization Phase 37
10.2.1. Double-blind Evaluation Period 37
10.2.2. Placebo wash-out period 40
10.2.3. Early Termination Evaluations 41
10.3. Study Restrictions 41
11. ASSESSMENT OF EFFICACY 42
11.1. Objective Assessments 42
11.2. Subjective Assessments 42
12. ASSESSMENT OF SAFETY 44
12.1. Safety Parameters 44
12.1.1. Laboratory Evaluations 44
12.1.2. Additional Laboratory Evaluations 45
12.1.3. Vital Signs and Body Measurements 45
12.1.4. Medical History and Physical Examinations 46
12.1.5. Electrocardiograms (ECGs) 46
12.1.6. Pregnancy 46
12.2. Definitions Related to Safety 47
12.2.1. Adverse Event 47
12.2.2. Serious Adverse Event 47
12.2.3. Adverse Event Follow-up 48
12.2.4. Adverse Event Reporting Period 48
12.2.5. Pre-existing Condition 48
12.3. Relationship to Study Drug 48
12.4. Recording Adverse Events 49
12.4.1. Adverse Events during Study Period 49

12.4.2. Post-study Adverse Event 49
12.4.3. Abnormal Laboratory Values 49
12.5. Reporting Adverse Events 50
12.5.1. Study Sponsor Notification by Investigator 50
12.5.2. EC/IRB Notification by Investigator 50
12.6. Unblinding Procedures 51
13. PHARMACOGENOMIC ASSESSMENT 52
14. STATISTICS 53
14.1. Statistical Methods 53
14.1.1. General 53
14.2. Sample Size and Accrual 54
14.3. Interim analysis 54
14.4. Patient Populations for Analysis 54
14.5. Pooling of Centers 55
14.6. Demography and Other Baseline Data 55
14.7. Study Medication 55
14.8. Concomitant Therapy 56
14.9. Efficacy Data 56
14.9.1. Efficacy Outcomes 56
14.9.1.1. Primary Efficacy Outcome 56
14.9.1.2. Secondary Efficacy Outcomes 56
14.9.2. Efficacy Analysis 57
14.9.2.1. General Data Analysis Considerations 57
14.9.2.2. Statement of the Null and Alternate Hypotheses 57
14.9.2.3. Analysis of the Primary Endpoint 57
14.9.2.4. Analysis of the Secondary Endpoints 57
14.9.2.5. Exploratory Efficacy Analyses 58
14.9.2.6. Graphs of Efficacy 58
14.10. Safety Data 58
14.10.1. Adverse Events 58
14.10.2. Laboratory Data 58
14.10.3. Vital Signs and Body Measurements 59
14.10.4. Electrocardiogram (ECG) 59
15. DIRECT ACCESS TO SOURCE DOCUMENTS 60

15.1. Definition of Source Document 60
15.2. Study Monitoring 60
15.3. Audits and Inspections 60
16. QUALITY CONTROL AND QUALITY ASSURANCE 62
16.1. Data Collection 62
16.2. Clinical Data Management 62
16.3. Database Quality Assurance 62
17. ETHICS 63
17.1. Ethics Review 63
17.2. Ethical Conduct of the Study 63
17.3. Written Informed Consent 63
18. DATA HANDLING AND RECORD KEEPING 65
18.1. Retention of Records 65
19. ADMINISTRATIVE PROCEDURES 66
19.1. Changes to the protocol 66
19.2. Periodic Reports to IRB/EC 66
19.3. Discontinuation of Study 66
19.4. Publication of Results 66
19.5. Investigator Agreement 68
20. REFERENCES 69
21. APPENDICES 70
21.1. Laboratory Ranges Used to Identified Clinically Notable Abnormal Laboratory Values 70
21.2. Vital Signs Values 71
21.3. Pharmacogenomic Sub-study Protocol 72
21.3.1. Introduction 72
21.3.2. Objectives 72
21.3.3. Study Design 72
21.3.4. Patient Selection and Withdrawal 73
21.3.5. Study Procedures 73
21.3.6. Statistical Analysis 73
21.3.7. Data Handling and Confidentiality 73
21.3.8. Ethical Considerations 74
21.3.9. Publication Plan 74

LIST OF TABLES

Table 1: Abbreviations and specialist terms 11

Table 2: Schedule of Evaluations 24

Table 3: Clinical Laboratory Tests 44

Table 4: SAE Criteria and Definitions 47

Table 5: SAE Reporting Information 50

3. LIST OF ABBREVIATIONS AND DEFINITIONS OF TERMS

Table 1: Abbreviations and specialist terms

Abbreviation	Description
AE	Adverse Event
AHI	Apnea-Hypopnea Index
ALT	Alanine Aminotransferase
ANOVA	Analysis of Variance
ANCOVA	Analysis of Covariance
AST	Aspartate Aminotransferase
AUC	Area Under the Curve
AUC (TAU)	The Area Under the Plasma Concentration-time Curve from Time ‘0’ to time ‘T’
BMI	Body Mass Index
β-HCG	Beta-human chorionic gonadotropin
BMS	Bristol Meyers Squibb
bpm	Beats per Minute
BUN	Blood Urea Nitrogen
BWSQ	Benzodiazepine Withdrawal Symptom Questionnaire
C	Celsius
CFR	Code of Federal Regulations
cm	Centimeter
C_{max}	The highest observed plasma concentration
C_{min}	The minimum observed plasma concentration
CNS	Central Nervous System
COPD	Chronic Obstructive Pulmonary Disease
CRF	Case Report Form
CRO	Clinical Research Organization
CRSD	Circadian Rhythm Sleep Disorders
DHHS	Department of Health and Human Services

Abbreviation	Description
dL	Deciliter
DLMO	Dim Light Melatonin Onset
dPSG	Diagnostic Polysomnography
DSM-IV	Diagnostic and Statistical Manual of Mental Disorders – Fourth Edition
DSST	Digit Symbol Substitution Test
EC	Ethical Committee
ECG	Electrocardiogram
e.g.	For example
EOS	End of Study
ET	Early Termination
F	Fahrenheit
FDA	Food and Drug Administration
GABA	Gamma-aminobutyric Acid
GCP	Good Clinical Practice
GGT	Gamma glutamyl transferase
HDPE	High density polyethylene
HIPAA	Health Insurance Portability and Accountability Act of 1996
hr	Hour
ICH	International Conference on Harmonization
ID	Identification
i.e.	In other words
IND	Investigational New Drug Application
IRB	Institutional Review Board
IU	International Unit
IUD	Intrauterine device
IV	Intravenous
IVRS	Interactive Voice Response System
kg	Kilogram
KSS	Karolinska Sleepiness Scale

Abbreviation	Description
L	Liter
LDH	Lactate Dehydrogenase
LPS	Latency to persistent sleep
MEL_{max}	The time when melatonin production reached 25% of the maximum melatonin concentration
mg	Milligram
min	Minute
mL	Milliliter
mm	Millimeter
mmHG	Millimeters of mercury
No.	Number
NREM	Non-rapid eye movement
o/n	Overnight
OTC	Over the Counter
oz	Ounce
PBO	Placebo
PE	Physical Examination
PER3	Period homolog 3
pH	Hydrogen ion concentration
PG	Pharmacogenomics
PHI	Protected Health Information
PLMAI	Periodic Limb Movement with Arousal Index
Pre-SQ	Pre sleep questionnaire
PSG	Polysomnography
PSQ	Post sleep questionnaire
RBC	Red blood cell
REM	Rapid eye movement
SAE	Serious Adverse Event
SAP	Statistical Analysis Plan
SE	Sleep Efficiency

Abbreviation	Description
SGOT	Serum Glutamic-Oxaloacetic Transaminase (also known as AST)
SGPT	Serum Glutamic Pyruvic Transaminase (also known as ALT)
SOC	System Organ Class
SOPs	Standard Operating Procedures
$T_{1/2}$	Time required for the plasma drug concentration to decrease by one half
T_{max}	Time to reach Cmax
TSH	Thyroid-Stimulating Hormone
TST	Total Sleep Time
µg	Microgram
U.S.	United States
VAS	Visual Analog Scale
WASO	Wake After Sleep Onset
WBC	White Blood Cell
WOCBP	Women of Childbearing Potential

4. INTRODUCTION

This document is a protocol for a human research study. This study is to be conducted according to United States (U.S.) and international standards of Good Clinical Practice (GCP) (Food and Drug Administration [FDA] and International Conference on Harmonization [ICH] guidelines), applicable government regulations, and Institutional research policies and procedures.

4.1. Background

Disorders of sleep and wakefulness which as a group are reported to chronically affect about 50 to 70 million Americans(1) comprise many distinct conditions. Insomnia is the most common sleep disorder. Insomnia is defined as the difficulty in initiating or maintaining sleep or experiencing nonrestorative sleep, and it is associated with next day consequences. Insomnia places a significant toll on individuals (impairs the individual's functioning, has a negative impact on individual's quality of life, and increases risks of accidents) and on society (increases health care costs and decreases job performance)(2). Even though the exact prevalence of insomnia is hard to determine since most information concerning the disorder has been obtained through questionnaires and interview surveys that address general difficulty in sleeping without regard to clinical significance or causal factors, it has been reported that insomnia affects approximately 10 to 17% of the American population (3;4). Insomnia is both a symptom (secondary to a medical, psychiatric, circadian, or sleep disorder) and a syndrome (primary insomnia that can not be attributable to other conditions). It is estimated that 25% of chronic insomnia is due to primary insomnia (3).

Prescription medications that are currently available to treat insomnia improve sleep by reducing sleep latency, increasing the total amount of sleep, and/or reducing the amount of awakening after sleep initiation. Benzodiazepines (e.g. Dalmane®, Halcion®, and Restoril®) are the oldest class of prescription drugs for the treatment of insomnia. Benzodiazepines work by enhancing the binding of the inhibitory GABA (gamma-aminobutyric acid) neurotransmitters which are thought to play the pivotal role within the sleep-inducing and maintenance systems(5). These compounds produce profound effects on the central nervous system (CNS), including residual daytime sedation, rebound insomnia, tolerance, amnesia, dependence, and cognitive and psychomotor impairment. Benzodiazepines also alter the sleep architecture(5).

A newer class of agents, known as non-benzodiazepine agents (e.g. Ambien®; Sonata®; Lunesta®) are becoming a more frequent and popular choice in the treatment of insomnia. These agents still promote sedation through the GABA receptors but the distinct specificity to the different GABA receptor subtypes confers them with different pharmacologic profiles and clinical activity(5). For example, Ambien® and Sonata® act selectively on the benzodiazepine omega-receptor subtype(6-8), are rapidly absorbed and well tolerated, but they are still only approved for the short-term treatment of insomnia. Lunesta®, on the other hand, interacts with GABA receptor complexes at binding domains located close to or allosterically coupled to benzodiazepine receptors (9). Lunesta® is the first sedative-hypnotic that has been approved without being restricted to short-term use.

A new class of agents that targets the melatonin receptors has recently been introduced. VEC-162 belongs to this class of agents. Because of its direct association with sleep and its involvement in the control of the circadian rhythm, melatonin is an obvious candidate for the treatment of sleep disorders. VEC-162 is a specific and potent agonist of the human MT1 and MT2 receptors which mediate melatonin's effect in the regulation of the sleep-wake cycle. In addition to the chronobiotic effect, melatonin has a soporific effect[10;11]. The exact mechanism by which melatonin exerts its soporific effect is not clear. It has been suggested that melatonin's soporific effect results from melatonin's ability to induce hypothermia[12].

To date, Vanda has trial data from one Phase II study (VP-VEC-162-2101) and one Phase III study (VP-VEC-162-3101) that show VEC-162's effectiveness in treating the symptoms of insomnia as well as circadian rhythm sleep disorders (CRSD). VP-VEC-162-2101 studied the safety and efficacy of VEC-162 in healthy volunteers in a 5-hour phase advance protocol. This study showed that VEC-162, when compared with placebo, induced a phase advance shift in the sleep-wake cycle on the first night of treatment in a dose-dependent manner. In addition, it also demonstrated an exposure-response relationship for sleep onset and sleep maintenance improvement, as measured by latency to persistent sleep (LPS) and wake after sleep onset (WASO), respectively. VP-VEC-162-3101 studied the safety and efficacy of VEC-162 in healthy volunteers in which transient insomnia has been induced by a circadian challenge (5-hour bedtime advance) and environmental stress (first night effect in a sleep laboratory). In this study, VEC-162 was able to reduce sleep latency (LPS), improve sleep maintenance (WASO), and increase sleep duration (TST).

On the basis of its pharmacological properties and preliminary experience in subjects, VEC-162 may be a safe and effective treatment for insomnia and CRSD since both disorders reflect an inability to initiate and maintain sleep of satisfactory duration, efficiency, or quality.

4.2. Relevant Data Summary

Details of nonclinical and clinical studies are provided in the Investigator Brochure.

4.3. Clinical Data

The clinical development of VEC-162 was initiated by Bristol Meyers Squibb (BMS) in 1997. BMS conducted five Phase I/II studies to develop the compound primarily for the treatment of insomnia. Vanda Pharmaceuticals Inc. in-licensed VEC-162 in 2004 and continued its clinical development. To that end, Vanda has conducted two clinical pharmacology studies (VP-VEC-162-1101, VP-VEC-162-1102), a Phase II proof of concept study (VP-VEC-162-2101) and a phase III study in a model of transient insomnia (VP-VEC-162-3101). A thorough QT study (VP-VEC-162-1103) is currently on-going. The data from the thorough QT study will be available prior to the first patient being dosed in this study. Depending upon the results of the thorough QT study, modifications to the ECG assessments performed in this study may be made.

4.3.1. VP-VEC-162-2101

Study VP-VEC-162-2101 was a randomized, double-blind, parallel-group, placebo-controlled study. The study consisted of a 2 to 4-week outpatient screening period

followed by an 8-day inpatient stay. Subjects were required to go to bed 5 hours earlier than their habitual bedtime (a 5-hour phase advance) on Day 4 of the inpatient stay. The primary objectives of this study were to investigate the exposure-response to VEC-162 on induction of an advance in the circadian melatonin rhythm as measured by Dim Light Melatonin Onset (DLMO), to investigate the exposure-response to VEC-162 on mean sleep efficiency parameters as measured by PSG, to investigate the exposure-response to VEC-162 on subjective sleepiness as measured by Karolinska Sleepiness Scale (KSS), to investigate the exposure-response to VEC-162 on objective neurobehavioral performance lapses during scheduled work-time as measured by computerized continuous performance testing, and to assess the safety and tolerability of VEC-162. Forty-five healthy volunteers, men and women aged 18 to 50, were enrolled into this study. Thirty-nine subjects were randomized. The order of the treatments was determined by a blinded randomization schedule. VEC-162 was able to significantly shift dim light melatonin onset compared with placebo, in a dose-dependent manner. VEC-162 was also able to minimize the disruption in full night sleep efficiency between Night 4 (first day of treatment) and Night 2 (baseline) in a dose-dependent manner. In addition, VEC-162, when compared to placebo, was able to reduce LPS on the first night of treatment (Night 4) when compared to baseline (Night 2). 100 mg of VEC-162 significantly reduced WASO. This study was not powered to detect differences in the subjective measurements (Karolinska Sleepiness Scale and Visual Analog Mood Scale) or in the performance test (Psychomotor Vigilance Task) and none were found. There were no deaths or SAEs. There was 1 discontinuation due to an AE, however, it was deemed unrelated to study medication by the Investigator. The most frequently reported treatment-emergent AEs in all groups in this study were decreased hematocrit levels (n = 18), decreased hemoglobin levels (n = 10), somnolence (n = 16), and headache (n = 7). The prevalence of these AEs was similar between VEC-162–treated subjects and placebo-treated subjects.

4.3.2. VP-VEC-162-3101

Study VP-VEC-162-3101 was a randomized, multicenter, double-blind, parallel-group, placebo-controlled study. This study was designed to evaluate the efficacy and safety of a single dose of VEC-162 (20, 50, or 100 mg) or placebo in healthy volunteers (aged 21 to 50) taken 30 minutes prior to their 5-hour phase-advanced bedtime. This study used both the first night effect and a 5-hour phase advance to induce transient insomnia in healthy subjects and to allow an appropriate comparison between VEC-162 and placebo. Four hundred and twelve (412) healthy subjects were randomized. VEC-162 was able to reduce latency to persistent sleep at all dose levels ($p=0.001$, $p<0.001$, and $p<0.001$ for 20, 50, and 100 mg, respectively), improve wake-after-sleep onset at the 20 and 50 mg dose ($p=0.026$ and $p=0.002$, respectively), and increase sleep efficiency for all doses when sleep was disturbed by a circadian challenge (5-hour bedtime advance) and environmental stress (first night effect in a sleep laboratory). VEC-162 demonstrated sleep onset and maintenance effects in this model of transient insomnia. Given the combined first night effect and circadian challenge in this study, efficacy of VEC-162 may reflect the combined soporific and circadian effects of VEC-162. In this study there were no deaths, SAEs, or discontinuations due to AEs. The most frequent (1% or more incidence overall) reported treatment emergent AEs in both the placebo and active groups

were nausea and headache. The incidence of nausea and headache was similar between VEC-162–treated subjects and placebo-treated subjects.

5. TRIAL OBJECTIVES AND RATIONALE

5.1. Objectives

5.1.1. Primary Objective

1. To assess the effects of 20 and 50 mg VEC-162 on the average latency to persistent sleep (LPS) as measured by polysomnography (PSG) on Nights 1 and 8.

5.1.2. Secondary objectives

1. To assess the effects of 20 and 50 mg VEC-162 on the average Wake After Sleep Onset (WASO), total sleep time (TST), and sleep efficiency (SE) as measured by PSG on Nights 1 and 8.
2. To assess the maintenance of effect of oral doses of 20 and 50 mg VEC-162 on the average LPS, WASO, TST, and SE as measured by PSG on Nights 22 and 29.
3. To assess the effects of 20 and 50 mg VEC-162 on PSG parameters for each individual evaluation night.
4. To assess the effects of single and multiple oral doses of 20 and 50 mg VEC-162 on subjective assessments such as sleep quality, depth of sleep, sleep latency, total sleep time, daytime function and daytime alertness.
5. To assess the next day residual effects of oral doses of 20 and 50 mg VEC-162 as measured by digit symbol substitution test (DSST) and visual analog scale (VAS).
6. To examine the potential rebound effects upon abrupt treatment cessation after 5 weeks of VEC-162 as measured by PSG.
7. To explore the safety and tolerability of multiple oral doses of 20 and 50 mg of VEC-162.
8. To explore subjective withdrawal symptoms after 5 weeks of VEC-162 treatment as measured by benzodiazepine withdrawal symptom questionnaire (BWSQ).
9. To explore the contribution of a polymorphism in the PER3 gene on the primary and secondary efficacy endpoints.

5.2. Rationale

5.2.1. Rationale for Dose and Schedule Selection

The oral doses selected are based on the efficacy data from VP-VEC-162-3101. This study showed significant improvement in both sleep onset and maintenance at 50 mg and 20 mg of VEC-162. Single oral doses of 1 mg to 300 mg were well tolerated by healthy subjects in previous clinical studies. In addition, administration of 1 mg to 150 mg of VEC-162 has been well tolerated by healthy volunteers for up to 28 consecutive days. Therefore, the highest dose of 50 mg is well within the established safety margin.

VP-VEC-162-3104 will evaluate the efficacy and safety of doses of VEC-162 (20 mg or 50 mg) or placebo taken in male and female patients aged 18 to 64 suffering from primary insomnia. For maximum efficacy, it is hypothesized that the time of maximum plasma concentration of VEC-162 should coincide with the time that patients go to bed. The peak C_{max} of VEC-162 is reached at 0.5-1 hour. Consequently, study medication will be taken 30 minutes prior to lights off (sleep clinic)/planned bedtime (outpatient).

5.2.2. Rationale for Study Design

Randomization is used to avoid any potential bias in the assignment of patients to treatment arms and to increase the likelihood that unknown and known patient characteristics (e.g. demographics) are evenly balanced across the different arms. Double-blind treatment is used to reduce potential bias in dosing, therapeutic decisions, and data handling. Single-blind (patients are blinded) treatment is used to reduced potential bias in the patient's perspective of the treatment effect. A parallel-group design is being used to allow the direct comparison across treatment groups with minimal confounding of time and to reduce the potential of sleep parameters improving from baseline merely due to the fact that patients are being enforced to follow better sleep hygiene practices during the course of the study. A fixed-dose design is used to allow an evaluation of the dose-response relationship.

The effect of VEC-162 on VAS and DSST will be assessed because next-day cognitive performance and alertness are important safety considerations for a sleep-promoting agent. In addition, a single-blind placebo wash-out will be done at the end of the 35-day chronic administration of study medication to assess any potential withdrawal or rebound insomnia effect of VEC-162.

6. INVESTIGATIONAL PLAN

6.1. Overall Study Design and Plan: Description

This is a multicenter, randomized, double-blind, placebo-controlled, parallel study to investigate the efficacy and safety of VEC-162 and matching placebo in male and female patients with primary insomnia. The study will be divided into 2 phases: the pre-randomization phase and the randomization phase. The pre-randomization phase consists of the screening visit and a one week single-blind placebo lead-in that includes two consecutive nights of PSG assessments. The randomization phase consists of a 5 week double-blind evaluation period and a 1-night single-blind placebo wash-out period. During the double-blind evaluation period, patients will undergo four overnight visits [Nights 1, 8, 22 and 29 (± 2 days)] in which PSG will be assessed. Patients will return to the clinic on Night 36 for an additional night of PSG assessments with single-blind placebo treatment.

6.1.1. Pre-randomization Phase

Informed consent will be obtained from potential patients prior to any study procedures being performed.

Screening Visit

The screening visit (Visit 1) will occur between 12 to 21 days prior to the start of the evaluation period (Night 1). During this visit, informed consent will be obtained from potential patients and their eligibility will initially be assessed based on sleep and medical history, current medication use, physical examination (PE), vital signs, ECG, and clinical laboratory results. During this visit, the Investigator will determine whether the patient meets the diagnostic criteria of primary insomnia according to the DSM-IV criteria in addition to having complaints of an inability to fall asleep (subjective latency ≥ 45 minutes) and an inadequate total amount of sleep (≤ 6.5 hours of sleep/night) at least 3 nights/week one month prior to the visit. Upon completion of the screening visit, potential patients will be given a sleep diary and a post-sleep questionnaire (PSQ) to take home with them. Patients must answer the questions in the sleep diary every morning within one hour of waking up for at least 7 days prior to the placebo lead-in visit. Patients are to be instructed to answer the questions in the outpatient PSQ the morning of the next scheduled visit. Patients must return the completed diary to the site on the next scheduled visit.

Single-blind Placebo Lead-in Period

Those patients who fulfill the preliminary inclusion/exclusion criteria will be asked to return to the sleep clinic approximately 2.5 hours prior to their scheduled bedtime for the placebo lead-in. The scheduled bedtime in the sleep clinic will be calculated as 8 hours prior to the patient's typical wake times during his/her normal work week. For example, the scheduled bedtime of a patient who normally wakes up to get ready and go to work at

6:00am will be 10:00pm. Once the scheduled bedtime is determined for a given patient, the same scheduled bedtime will be used for that patient throughout all the inpatient sleep episodes.

The single-blind placebo lead-in period consists of two consecutive nights at the sleep clinic followed by approximately 5 days of outpatient treatment. At the beginning of the placebo lead-in, the sleep diary data from the seven consecutive days immediately preceding Visit 2 will be reviewed to confirm eligibility. The patient must have completed the sleep diary for at least 4 days out of the seven. On the first night of the placebo lead-in period, instructions and a practice session should be given to the patient on how to properly complete the DSST and VAS. These first practice measurements will not be used in the analysis of this study. In addition, the patients will take a breathalyzer test and complete the pre-sleep questionnaire (Pre-SQ) each inpatient night. Patients will complete the Pre-SQ prior to receiving single-blind placebo capsules 30 minutes (± 5 minutes) prior to lights off. Lights off should occur within 15 minutes of their scheduled bedtime.

PSG recordings will be conducted in each of the two consecutive nights of the single-blind placebo lead-in period. The first PSG will be a diagnostic PSG (dPSG) which will also be used to exclude patients with any other sleep disorder (e.g. sleep apnea). Patients will be awakened at the end of the 8-hour sleep episode and will be asked to complete a battery of tests 1 hour (± 15 min) after awakening. The battery of tests includes the following assessments **in order**: PSQ, VAS and DSST. On the morning after the dPSG, patients will be released from the sleep clinic and reminded to return to the sleep clinic that evening for a repeat night of testing.

Upon completion of the second night of PSG recording, patients will be given a bottle containing single-blind placebo capsules and will be instructed to take the study medication approximately 30 minutes before their planned bedtime every night until they return to the clinic. The definition of planned bedtime is the bedtime the patient is planning to go to sleep that particular evening given that there will be natural schedule variability from night to night in the outpatient setting. Furthermore, the patients will be notified that for their safety they should only take their study medication if they have at least a 6-hour opportunity to sleep and patients should confine their activities after taking study medication to those necessary to prepare for bed.

6.1.2. Randomization Phase

Double-blind Evaluation Period

On Night 1, patients should arrive to the sleep clinic approximately 2.5 hours prior to their scheduled bedtime for pre-dose safety assessments which include a urine pregnancy test for women of child-bearing potential (WOCBP), a urine drug test, a breathalyzer test, and an assessment of vital signs. Patients that continue to meet the eligibility criteria for this study will then be randomly assigned to 1 of 3 treatment groups: 20 mg VEC-162, 50 mg VEC-162, or placebo. Patients will be required to complete the pre-sleep questionnaire prior to study drug administration and will receive double-blind study medication 30 minutes (± 5 minutes) prior to lights off and PSG will be recorded for 8 hours. Lights off should occur within 15 minutes of their scheduled bedtime. Patients will be awakened at the end of the 8-hour sleep episode and will be asked to complete the battery of test (PSQ, VAS and DSST in this order) 1 hour (± 15 minutes) after

awakening. Prior to discharge from the sleep lab, patients will be dispensed study medication under double-blind conditions and instructed to take one capsule every night approximately 30 minutes prior to their planned bedtime. Furthermore, the patients will be notified that for their safety they should only take their study medication if they have at least a 6-hour opportunity to sleep. Patients will also be given a PSQ to take home with them. This outpatient PSQ should be completed by the patient on the morning of the next scheduled study visit. Patients will be released from the sleep clinic after all morning assessments have been completed.

Patients will be required to return to the sleep clinic on Nights 8, 22, and 29 (± 2 days) for additional nights of PSG recording. At the beginning of each study visit, patients will be required to take a breathalyzer test, a urine pregnancy test for WOCBP, and complete the pre-sleep questionnaire prior to study drug administration. They will receive double-blind study medication 30 minutes (± 5 minutes) prior to light off. Lights off should occur within 15 minutes of their scheduled bedtime. Patients will be awakened at the end of the 8-hour sleep episode and will be asked to complete the battery of tests (PSQ, VAS and DSST in this order) 1 hour (± 15 minutes) after awakening. At each of these visits, the patients will be dispensed enough medication to last until the next scheduled visit and will be reminded to take one capsule of study medication each night approximately 30 minutes prior to their planned bedtime. Patients will also be given a PSQ to take home with them and instructed to complete the outpatient PSQ on the morning of the next scheduled study visit. Patients will be allowed to leave the sleep clinic after completion of the morning assessments. In addition, on Day 30, patients will be asked to complete the Benzodiazepine Withdrawal Symptom Questionnaire (BWSQ) prior to completing the battery of tests.

Single-blind Placebo Wash-out Period

Patients will be requested to return to the clinic on Night 36 (± 2 days) approximately 2.5 hours prior to their scheduled bedtime for an additional night of PSG recording. Patients will have a breathalyzer test and complete the pre-sleep questionnaire prior to drug administration. They will receive single-blind placebo 30 minutes (± 5 minutes) prior to lights off and PSG will be recorded for 8 hours. Patients will be awakened at the end of the 8-hour sleep episode. End-of-study (EOS) assessments will be performed on the morning of Day 37 (or at the time of early discontinuation). EOS assessments include safety evaluations such as serum pregnancy test for females, physical examination (PE), vital signs, ECG, and clinical laboratory review. EOS assessments also include the completion of the BSWQ followed by the battery of tests (PSQ, VAS and DSST) 1 hour (± 15 minutes) after awakening. Patients will be released from the sleep clinic after all EOS assessments have been completed.

Table 2: Schedule of Evaluations

PHASE	PRE-RANDOMIZATION					RANDOMIZATION										ET
PERIOD	Screen	PBO Lead-in				Double-blind Evaluation								PBO Wash-out		
Day(s)		N -7	D -6	N -6	D -5	N1	D2	N8	D9	N22	D23	N29	D30	N36	D37 & EOS	
Visit	1	2*				3		4*		5*		6*		7*		
Informed Consent Form(s) [1]	X															
Patient Demography	X															
Inclusion/Exclusion criteria	X	X[2]				X[2]										
Medical/Sleep/Psychiatric History	X															
Vital Signs[3]	X	X	X		X	X	X		X		X		X		X	X
Owl-Lark Questionnaire	X															
Physical Examination	X					X[2]									X	X
ECG	X					3X							X		X	X
Labs (Hematology, Chemistry and Urinalysis)	X					X							X		X	X
Breathalyzer		X		X		X		X		X		X		X		
Urine Drug Screen	X	X				X				X						
Serum B-HCG (WOCBP)	X														X	X
Urine Pregnancy (WOCBP)		X[4]				X[4]		X[4]		X[4]		X[4]		X[4]		
Diagnostic PSG		X														
PSG				X		X		X		X		X		X		
Pre-sleep questionnaire		X		X		X		X		X		X		X		
PSQ, VAS and DSST		X[5]	X		X		X		X		X		X		X	
IVRS call[6]	X					X									X	X
Randomization						X										
PG blood sample[7]						X										
PER3 Genotyping						X										
BWSQ													X		X	
Study Medication		Daily; 30 min before light off/planned bedtime				Daily; 30 minutes before light off/planned bedtime								X		
Drug Dispensing for Outpatient Use[8]					X		X		X		X		X			
Dispense PSQ[9]	X				X		X		X		X		X			
Dispense Sleep Diary[10]	X															
Collect Outpatient PSQ		X				X		X		X		X		X		
Adverse Event Query		X	X	X	X	X	X	X	X	X	X	X	X	X	X	X
Prior/Concomitant Med	X	X		X		X		X		X		X		X	X	X

D= Day; N= Night; PBO= placebo; EOS= end of study; ET= early termination; WOCBP= women of childbearing potential; PSG= polysomnography; PG= Pharmacogenomic; BWSQ= Benzodiazepine Withdrawal Symptom Questionnaire; Med= Medication.
*These visits have a ± 2-day window. Visit 2 must have two consecutive nights of PSG assessment.
[1] The informed consent must be sign prior to any study procedure being done.
[2] Abbreviated- Only required to document changes.
[3]Body height will only be recorded at screening. Weight will only be recorded at screening, Night 1 and EOS.
[4]Pregnancy test must be negative prior to dosing.
[5]At Night -7, only the DSST and VAS will be administered. This session is for practice purpose only. Results will not be used in the analysis.
[6]The IVRS will be called to assign the patient ID and single-blind PBO bottle at the screening visit. The Investigator will call the IVRS to assign a double-blind medication kit on Night 1. The IVRS will also be called to report patient completion or discontinuation.
[7]Blood sample for PG assessments should only be obtained if patients sign the optional PG Informed Consent.
[8] Unused study drug should be collected from the patient and counted at the beginning of Visit 3, 4, 5, 6, and 7.
[9] Patients will be instructed to complete the PSQ the morning of the next scheduled PSG visit.
[10] The sleep diary must be completed for at least seven days and returned to the site on Visit 2.

7. SELECTION AND WITHDRAWAL OF PATIENTS

7.1. Patient Inclusion Criteria

Each patient must meet the following criteria for inclusion in the study:

1. Ability and acceptance to provide written informed consent;
2. Men or women between 18 – 64 years, inclusive;
3. Body Mass Index (BMI) of >18 and <33kg/m2 (BMI = weight (kg)/ [height (m)]2);
4. Women of child-bearing potential (defined as less than 1 year post-menopausal or not surgically sterile) must be using an acceptable method of birth control (e.g., oral contraceptives, steroids, patch, intrauterine device [IUD], diaphragm or condom with spermicidal jelly or foam or abstinence, surgical sterility, or cervical cap) for a period of 35 days before the first dosing and must have a negative pregnancy test at the screening and Night 1 visits;

   Note: Women using hormonal methods of birth control (e.g. oral contraceptives, patch, and steroids) must use an additional method of birth control during the study and for one month after the last dose.
5. Meets diagnostic criteria for primary insomnia as defined in DSM-IV;
6. Subjective sleep latency of ≥ 45 minutes at least 3 nights/week **and** subjective total sleep time ≤ 6.5 hours of sleep at least 3 nights/week based on sleep history (30 days prior to the screening visit);
7. Subjective sleep latency of ≥ 45 minutes at least 3 nights/week **and** subjective total sleep time ≤ 6.5 hours of sleep at least 3 nights/week based on sleep diary;
   a. Note: The data from the seven consecutive days immediately preceding Visit 2 must be used for this criterion. The patient must have completed the sleep diary for at least 4 days out of the seven.
8. A mean LPS of ≥ 30 minutes on two consecutive placebo lead-in PSG with no night having a LPS less than 20 minutes;
9. Habitual bedtime between 9:00 PM and 1:00 AM at screening for at least one month prior to screening;
10. No history or evidence of restless leg syndrome or periodic limb movement disorder based on a Periodic Limb Movement with Arousal Index (PLMAI) score >10 on diagnostic PSG;
11. No history or evidence of sleep apnea based on Apnea-Hypopnea Index (AHI) score >10 on diagnostic PSG;
12. Willing and able to comply with study requirements and restrictions.

7.2. Patient Exclusion Criteria

Patients will be excluded from the study if any of the following criteria apply:

1. History of drug or alcohol abuse as defined in DSM-IV, Diagnostic Criteria for Drug and Alcohol Abuse, within the 12 months prior to screening and/or regular consumption of alcoholic drinks (> 2 drinks/day or > 14 drinks/week);
2. A positive test for drugs of abuse at the screening visit or the first night of the single-blind period;
    a. Note: A positive drug screen at Visit 1 due to medication use needs to be discussed with the medical monitor on a case-by-case basis.
3. Evidence of recent alcohol consumption as determined by breathalyzer test;
4. Smoke more than 10 cigarettes/day and not willing to follow the smoking restrictions in this study or use tobacco products during nightly awakenings;
5. History of psychiatric disorders including Major Depressive Disorder, Generalized Anxiety Disorder and delirium, within the 12 months prior to screening;
6. History of chronic obstructive pulmonary disease (COPD), seizures, sleep apnea, narcolepsy, circadian-rhythm sleep disorder, parasomnia or any other sleep disorder other than chronic insomnia;
7. Traveled more than three time zones 2 weeks prior to the screening visit or plan to travel across more than two time zones during the study;
8. Worked night, rotating, or split (period of work, followed by break, and then return to work) shift work within 1 month of the screening visit or plan to work these shifts during the study;
9. History or current evidence of hypercholesterolemia or hypertension, unless currently controlled and stable with protocol allowed medication 30 days prior to the first day of single-blind study medication;
10. History or current evidence of cardiovascular, hepatic, hematopoietic, renal, gastrointestinal or metabolic dysfunction.
11. History of intolerance and/or hypersensitivity to melatonin or melatonin agonists, or anyone who has taken melatonin or any other prescription or OTC medication that affect the sleep-wake cycle within 5 days of the single-blind medication;
12. Pregnant or lactating females;
13. Indication of impaired liver function (i.e., values $\geq$ 2 times the upper limit of normal for at least two of the following- aspartate aminotransferase, alanine aminotransferase, and total bilirubin);
14. Two consecutive abnormal vital sign measurements during the first screening visit. The following vital sign measurements will be considered abnormal for this study:
    a. sitting systolic blood pressure of less than 90 mmHg or greater than 160 mmHG;

   b. a sitting diastolic blood pressure of less than 60 mmHg or greater than 100 mmHg;
   c. a heart rate less than 40 or greater than 95 bpm.

15. Exposure to any investigational drug within 30 days of screening, including placebo;
16. Participation in a previous BMS-214778/VEC-162 trial;
17. Use of any prescription, OTC medication or sleep aids including herbal or other nontraditional sleep-inducing preparation within 7 days (or 5 half-lives of the drug, whichever is longer) of the single-blind medication except for the acceptable medications detail in Section 8.2.
18. Any other sound medical reason as determined by the clinical investigator.

7.3. Patient Withdrawal Criteria

The term "discontinuation" refers to the patient's premature withdrawal from the study before completing all scheduled evaluations.

Patients may be discontinued from the study for any of the following reasons:

- If in the Investigator's judgment, continuation in the study may prove harmful to the patient. Such a decision may be precipitated by adverse events, including the occurrence of rash, changes in vital signs, physical examination, ECG, or laboratory tests. The Investigator will maintain autonomy in making medical/safety decisions regarding the patient's continued participation in the trial. Clinically notable abnormalities in vital signs or laboratory tests are provided in Appendices 21.2 and 21.1, respectively, to guide clinical focus regarding a patient's continued participation.
- Noncompliance
- At the request of the patient.

Any time a patient discontinues from the study, the interactive voice response system (IVRS) should be notified.

Documented reason: It will be documented whether or not each patient completed the clinical study. If, for any patient, study treatment or observations were discontinued, the reason will be recorded (only 1 choice is acceptable) on the electronic case report form (electronic CRF). Acceptable reasons for a patient discontinuing participation in this clinical study are as follows:

1. protocol deviation (including noncompliance)
2. adverse event(s)(including abnormal laboratory values, abnormal test procedures, and pregnancy)
3. lost to follow-up
4. death
5. patient withdrew consent

6. unsatisfactory therapeutic effect
7. other (specify)

Patients who prematurely discontinue from the study (Early Termination) will undergo a complete safety assessment as detailed in the Schedule of Evaluation (see Table 2). Patients who discontinue because of an AE, abnormal laboratory value, or abnormal test result will be followed until resolution or for 30 days, whichever is less. If a patient elects to withdraw from the study, he/she will be informed that it is extremely important that the reason for discontinuation be reported and that all EOS assessments required by the protocol are performed.

8. TREATMENT OF PATIENTS

8.1. Dosing

This section describes the dosing schedule for the Lead-in, the Evaluation, and the Wash-out Periods.

During the Lead-in Period:

- A single oral dose of placebo will be administered to the patients on Visit 2 thirty minutes (± 5 minutes) prior to lights off under single-blind conditions. The placebo capsule will be taken with water. Patients will continue to ingest one placebo capsule (single-blind) every night approximately 30 minutes prior to their planned bedtime until Night 1. Patient will take the medication with water.

During the Double-Blind Evaluation Period:

- On Visits 3-6, study medication will be dispensed by study personnel under double-blind conditions. Patients will take oral doses of 20 mg VEC-162, 50 mg VEC-162 or placebo (blinded) 30 minutes (± 5 minutes) prior to lights off. Capsules will be taken with water.
- While at home, patients will continue to take oral doses of either 20 mg VEC-162, 50 mg VEC-162 or placebo approximately 30 minutes before planned bedtime. Capsules will be taken with water. Patients and study personnel will remain blinded as to the contents of the bottles.

During the Wash-out Period:

- A single oral dose of placebo will be administered to the patients on Visit 7 (Night 36 ±2 days) 30 minutes (± 5 minutes) prior to lights off under single-blind conditions. The placebo capsule will be taken with water.

8.2. Concomitant Medications

All concomitant illnesses will be treated in accordance with prevailing medical practice. The study staff will record the names of all drugs prescribed, duration of usage, and indication on the Concomitant Medication electronic CRF.

The following drugs (drug classes) are permitted during the study:

- Ibuprofen or acetaminophen
- Birth control medication
- Colace (Docusate Sodium)
- Fiber laxatives

- Vitamins and supplements (except ones with CNS or sedative effects or those that can alter the sleep-wake cycle)
- Baby aspirin
- Lactaid®
- The following statins: fluvastatin (Lescol®), pravastatin (Pravachol®), and rosuvastatin (Crestor®)
- Anti-Hypertensive medication except for the following drugs or drug classes:
  - Calcium channel blockers such as amlodipine, felodipine, isradipine, mibefradil, nicardipine, nifedipine, nimodipine, nisoldipine, diltiazem, verapamil
  - Others: Eplerenone, Ambristentan, alliskiren, Amlodipine Besylate/Benazepril, Hydrochloride, Amlodipine Besylate/Valsartan, Bisoprolol fumarate/hydrochlorothiazide, bosentan, Candesartan Cilexetil/Hydrochlorothiazide, diazoxide
- Topical steroids
- Saline nasal spray
- Saline eye drops
- Additional medications may be permitted on a case-by-case basis if pre-approved by the medical monitor

8.3. Treatment Compliance

Compliance at the clinical site during inpatient visits will be assured by administration of the study drug under the supervision of a member of the study team. Compliance in this study will be assessed by the Investigator. The Investigator will consider capsule use (returned capsules) and feedback from study staff when assessing compliance. An overall assessment of compliance will be documented in the CRFs. Patients with a compliance of less than 80% will be re-educated by the Investigator or study staff on the importance of taking the study medication properly.

Any overdose that occurs during study participation must be reported to Covance Drug Safety Services (Vanda's designee) using the Study Drug Overdose Report Form.

8.4. Treatment Assignment and Randomization

8.4.1. Treatment Assignment

Patient Identification (ID)

Each patient who signs an informed consent form must receive a patient identification number. Patient identification numbers will be assigned during the screening visit by calling the IVRS for the next available patient number. This patient identification

number will remain the same throughout the study, and will be used by the site to identify the patient. The patient identification number (ID) consists of a 3-digit site number (Site No.) and a 4-digit patient number (Patient No.). The Site No. and Patient No. will be separated by a hyphen. This 8 digit alpha numeric identifier will be manually entered in the "Patient ID" space on the electronic CRF for a given patient.

Site No. - Vanda will assign a unique, 3-digit number to each site.

Patient No. - The IVRS will assign a 4-digit number to each patient in sequential order by site at the time of the screening visit call. The first (leftmost) will be assigned "1." Example: The first patient who signs a consent form at Site 301 will be assigned the ID 301-1001, the second, 301-1002, etc.

The ID of a patient who discontinues from the study for any reason after having been assigned an ID will not be reassigned. A patient's ID number will remain the same for that patient throughout all phases of the study.

8.4.2. Randomization

The randomization will be performed using the IVRS, a validated system that automates the random assignment of treatment groups to randomization numbers. The randomization scheme will be reviewed and approved by a biostatistician designated by Vanda. On Night 1, the Investigator or designee will call the IVRS to assign a randomization number which will determine which study medication the patient will be assigned.

9. STUDY DRUG MATERIALS AND MANAGEMENT

9.1. Study Drug

The investigational drug will be 20 mg and 50 mg VEC-162 capsules for oral administration. All capsules will be size 1 and the color will be white opaque. To preserve the study blinding, the placebo capsule will be identical in size, shape, color, and appearance to the VEC-162 capsules.

9.2. Study Drug Packaging and Labeling

Study medication will be provided in HDPE bottles with child-resistant caps containing polyester cushioning and desiccant.

Pre-randomization Phase

The single-blind placebo for the lead-in period will be provided as individual bottles identified by a unique medication number. Each bottle will contain 10 placebo capsules and the bottle will have a two-part tear-off label. The second part of the label will be torn off at the site and affixed to the patient's source documents before the bottle is dispensed to the patient. Medication labels will comply with US legal requirements and be printed in English. At the screening visit, the IVRS will assign a bottle of single-blind study medication to the patient for the pre-randomization phase of the study.

Randomization Phase

After eligibility is verified, the Investigator is to call the IVRS on Night 1 to assign a randomization number and medication kit to the patient for the randomization phase of the study. A kit containing 6 bottles of study medication labeled bottle # 1-6 will be assigned to a specific patient. Each bottle within the kit will be labeled with the same medication number. Bottles # 1-5 will contain 10 capsules of 20 mg, or 50 mg of VEC-162 or placebo to provide enough medication for one week of treatment. Bottle # 6 will contain 10 placebo capsules that will be used during the overnight single-blind placebo wash-out.

At each visit, a new bottle of study medication will be used. While at the sleep lab, administration of the study drug will be under the supervision of a member of the study team. The next morning, the bottle(s) will be dispensed to the patient for study medication to last until the next schedule visit. Bottles must be dispensed <u>in consecutive order</u> beginning with bottle #1 (i.e. bottle #1 will be used during and dispensed to the patient at the end of Visit 3, bottle #2 will be used during Visit 4 and bottles #2 and #3 will be dispensed to the patient at the end of Visit 4, bottle #4 will be used during and dispensed to the patient at the end of Visit 5, bottle #5 will be used during and dispensed to the patient at the end of Visit 6, bottle #6 will be used during Visit 7). Patients should be instructed to bring all bottles along with any unused capsules to the study site at his/her next visit.

Each kit will be identified by a unique medication number and have a two-panel blinded label which will comply with the legal requirements of the United States and will be printed in English. The two panel labels on each kit of study medication will have a tear-off portion which must be removed and affixed to the patient's source documents when the kit is allocated to the patient. Each of the 6 bottles in each kit will also have a two-panel blinded label displaying the same medication number as the kit which must also be removed and affixed to the patient's source documents when the bottle is given to the patient.

9.3. Study Drug Storage

Drug supplies must be stored in a safe, secure location at 20°C to 25°C (68°F to 77°F), and must be protected from light, heat, and moisture. The storage conditions for study medication will be described on the medication label.

9.4. Study Drug Accountability

Vanda Pharmaceuticals Inc. is responsible for assuring that the quality of the study drug is adequate for the duration of the study.

Study medication should be used in accordance with the protocol, under the supervision of the Investigator or delegated by the Investigator to the site pharmacist or other personnel trained to store and dispense investigational drugs.

The Investigator or a designee is responsible for taking an inventory of each shipment of study medication received and comparing it with the accompanying packing list. The Investigator or a designee will verify the accuracy of the information on the packing list, sign and date the acknowledgement of receipt, retain a copy in the study file, and send a copy to Vanda or its designee.

The Investigator or Co-Investigator(s) must agree to supply study medication only to patients enrolled in the study. Each patient must be given the study medication corresponding to his/her medication code number. It is the responsibility of the Investigator to ensure that a current record of study drug disposition is maintained. Records or logs must comply with applicable regulations and guidelines, and should include:

- Amount of study drug received
- Medication batch number
- Dates of drug inventory movement
- Unique patient identifier
- Amount dispensed to each patient
- Amount returned by each patient
- Initials of person responsible for each drug inventory entry

Accurate recording of all study medication administration (including dispensing and dosing) will also be made in the appropriate section of the patient's electronic CRF and source documents.

Vanda or its designee will instruct the Investigator on the return or destruction of unused study medication. If any study medication was lost or damaged, its disposition should be documented in the patient's source documents as well as the drug accountability record. Study medication supplies will be retained at the clinical site until instructions for return or destruction of the supplies are received from Vanda or its designee.

10. STUDY ASSESSMENTS AND RESTRICTIONS

10.1. Pre-Randomization

10.1.1. Screening Visit

The following evaluations will be performed **after** the patient signs the informed consent form:

Visit 1 (12 to 21 days prior to Night 1)

- IVRS call to assign patient ID and a medication bottle
- Prior/current medication
- Collection of patient's demographic information (e.g. date of birth, race, gender)
- Inclusion/exclusion criteria
- Medical, sleep and psychiatric history
- Owl-lark questionnaire
- Vital signs (body temperature, respiratory rate, blood pressure, pulse, weight, and height)
- Full physical exam
- Laboratory evaluations (hematology, biochemistry and urinalysis)
- Full standard 12-lead ECG
- Urine drug screen
- Serum β-HCG for women of childbearing potential (WOCBP), except those who have been postmenopausal for at least 1 year or are surgically sterile
- Dispense outpatient PSQ
- Dispense sleep diary

10.1.2. Single-blind Placebo Lead-in Period

Visit 2 (7 days ± 2 prior to Night 1)

Night -7

- Review of sleep diary (only data from 7 days immediately preceding Visit 2)
- Collect outpatient PSQ
- Review of inclusion-exclusion criteria
- Vital Signs (body temperature, respiratory rate, blood pressure, and pulse)
- Practice for battery of tests (VAS, and DSST, in this order)

- Breathalyzer
- Urine drug screen
- Urine pregnancy test for WOCBP, except those who have been postmenopausal for at least 1 year or are surgically sterile (result must be negative prior to dosing)
- Pre-sleep questionnaire
- Adverse event query
- Review of concomitant medication
- Administration of single-blind treatment (30 ± 5 minutes prior to light off)
- Diagnostic PSG (score locally for exclusionary purpose; score will be confirmed by central scoring facility)

Day -6

- Vital Signs (body temperature, respiratory rate, blood pressure, and pulse)
- Adverse event query
- Battery of tests (Post-sleep questionnaire, VAS, and DSST, in this order; one hour [± 15 minutes] after awakening)

Night -6

- Breathalyzer
- Pre-sleep questionnaire
- Adverse event query
- Review of concomitant medication
- Administration of single-blind treatment (30 ± 5 minutes prior to light off)
- PSG

Day -5

- Vital Signs (body temperature, respiratory rate, blood pressure, and pulse)
- Adverse event query
- Battery of tests (Post-sleep questionnaire, VAS, and DSST, in this order; one hour [± 15 minutes] after awakening))
- Drug Dispensing for out-patient use (single-blind treatment)
- Dispense outpatient PSQ and instruct patient to complete prior to the next scheduled visit

Nights -5 (± 2 days) to -1; outpatient

- Take study medication 30 minutes prior to planned bedtime

10.2. Randomization Phase

10.2.1. Double-blind Evaluation Period

Visits 3-6

Night 1 (Visit 3)

- Review of inclusion-exclusion criteria
- Collect unused drug, from single-blind placebo lead-in
- Collect outpatient PSQ
- Brief Physical examination (conducted only if patient reports change in health or experiences an AE)
- Full standard 12-lead ECG in triplicate (approximately 5 minutes apart)
- Vital signs (body temperature, respiratory rate, blood pressure, pulse, and weight)
- Urine drug screen
- Urine pregnancy test for WOCBP, except those who have been postmenopausal for at least 1 year or are surgically sterile (result must be negative prior to dosing)
- Breathalyzer
- Randomization through IVRS to assign medication kit (**after** patient meet all inclusion exclusion criteria including negative pregnancy test)
- Laboratory evaluations (hematology, biochemistry and urinalysis)
- Blood draw for optional pharmacogenomic (PG) analysis (if consent is given by the patient)
- Blood draw for PER3 genotyping
- Pre-sleep questionnaire
- Review of concomitant medication
- Adverse event query
- Study medication administration (30 ± 5 minutes prior to light off)
- PSG

Day 2 (Visit 3)

- Vital Signs (body temperature, respiratory rate, blood pressure, and pulse)
- Adverse event query
- Battery of tests (Post-sleep questionnaire, VAS, and DSST, in this order; one hour [± 15 minutes] after awakening)

- Drug dispensing for out-patient use
- Dispense outpatient PSQ and instruct patient to complete prior to the next scheduled visit

Night 8 ± 2 days (Visit 4)

- Collect unused study drug and assess compliance
- Collect outpatient PSQ
- Urine pregnancy test for WOCBP, except those who have been postmenopausal for at least 1 year or are surgically sterile (result must be negative prior to dosing)
- Breathalyzer
- Pre-sleep questionnaire
- Review of concomitant medication
- Adverse event query
- Study medication administration (30 ± 5 minutes prior to light off)
- PSG

Day 9 (Visit 4)

- Vital Signs (body temperature, respiratory rate, blood pressure, and pulse)
- Adverse event query
- Battery of tests (Post-sleep questionnaire, VAS, and DSST, in this order; one hour [± 15 minutes] after awakening)
- Drug dispensing for out-patient use
- Dispense outpatient PSQ and instruct patient to complete prior to the next scheduled visit

Night 22 ± 2 days (Visit 5)

- Collect unused study drug and assess compliance
- Collect outpatient PSQ
- Urine drug screen
- Urine pregnancy test for WOCBP, except those who have been postmenopausal for at least 1 year or are surgically sterile (result must be negative prior to dosing)
- Breathalyzer
- Pre-sleep questionnaire
- Review of concomitant medication
- Adverse event query
- Study medication administration (30 ± 5 minutes prior to light off)

- PSG

Day 23 (Visit 5)

- Vital Signs (body temperature, respiratory rate, blood pressure, and pulse)
- Adverse event query
- Battery of tests (Post-sleep questionnaire, VAS, and DSST, in this order; one hour [± 15 minutes] after awakening)
- Drug dispensing for out-patient use
- Dispense outpatient PSQ and instruct patient to complete prior to the next scheduled visit

Night 29 ± 2 days (Visit 6)

- Collect unused study drug and assess compliance
- Collect outpatient PSQ
- Urine pregnancy test for WOCBP, except those who have been postmenopausal for at least 1 year or are surgically sterile (result must be negative prior to dosing)
- Breathalyzer
- Pre-sleep questionnaire
- Review of concomitant medication
- Adverse event query
- Study medication administration (30 ± 5 minutes prior to light off)
- PSG

Day 30 (Visit 6)

- Laboratory evaluations (hematology, biochemistry and urinalysis)
- Full standard 12-lead ECG
- Vital Signs (body temperature, respiratory rate, blood pressure, and pulse)
- Adverse event query
- BWSQ (prior to battery of test)
- Battery of tests (Post-sleep questionnaire, VAS, and DSST, in this order; one hour [± 15 minutes] after awakening)
- Drug dispensing for out-patient use
- Dispense outpatient PSQ and instruct patient to complete prior to the next scheduled visit

Every outpatient night during double-blind period

- Take study medication 30 minutes prior to planned bedtime

10.2.2. Placebo wash-out period

Visit 7

Night 36 ± 2 days

- Collect unused study drug and assess compliance
- Collect outpatient PSQ
- Urine pregnancy test for WOCBP, except those who have been postmenopausal for at least 1 year or are surgically sterile (result must be negative prior to dosing)
- Breathalyzer
- Pre-sleep questionnaire
- Review of concomitant medication
- Adverse event query
- Administration of single-blind treatment (30 ± 5 minutes prior to light off)
- PSG

Day 37 and End-of-study Evaluations

End-of-study procedures will be performed the morning of Day 37

- Full physical examination
- Full standard 12-lead ECG
- Vital signs (body temperature, respiratory rate, blood pressure, pulse, and weight)
- Laboratory evaluations (hematology, biochemistry and urinalysis)
- Serum pregnancy test for women of childbearing potential (WOCBP), except those who have been postmenopausal for at least 1 year or are surgically sterile
- BWSQ (prior to the battery of tests)
- Battery of tests (Post-sleep questionnaire, VAS, and DSST, in this order; one hour [± 15 minutes] after awakening))
- Adverse events query
- Review of concomitant therapy
- IVRS call to report patient completion

10.2.3. Early Termination Evaluations

Efforts should be made so that early termination procedures happen as soon as possible after discontinuation.

- Full physical examination
- Full standard 12-lead ECG
- Vital signs (body temperature, respiratory rate, blood pressure, pulse, and weight)
- Laboratory evaluations (hematology, biochemistry and urinalysis)
- Serum pregnancy test for women of childbearing potential (WOCBP), except those who have been postmenopausal for at least 1 year or are surgically sterile
- Adverse events query
- Review of concomitant therapy
- IVRS call to report patient discontinuation

10.3. Study Restrictions

- Patients may not consume any alcohol-containing beverages for 24 hours prior to reporting to a study visit where an overnight PSG will be performed. Patients will be checked with breathalyzers during all check-in procedures. If patients test positive for alcohol, the patients will not be able to continue in the study.
- Consumption of xanthine containing beverages or food and beverages (e.g., coffee, tea, soda, and chocolate) is not permitted after 1:30 PM while participating in this study. If a deviation occurs the day of an overnight PSG assessment, it must be noted in the source documents and on the electronic CRF.
- Patients are to refrain from strenuous exercise and contact sports (e.g., weight training, aerobics, and football) for 24 hours before a study visit where blood will be drawn and for 4 hours prior to reporting to a study visit where an overnight PSG will be performed.
- Patients are not permitted to smoke more than 10 cigarettes/day or use tobacco products during nightly awakenings.
- Patients should not travel more than 2 time zones during the study.

11. ASSESSMENT OF EFFICACY

11.1. Objective Assessments

- PSG
- DSST

Polysomnography (PSG)

Polysomnography is an overnight recording of sleep pattern and behaviors associated with sleep. A variety of sensors are applied with paste or tape to the body's surface to record brain waves, eye movements, muscle tone, body movements, heart rate, and breathing. The recording of the sensors can be helpful in determining sleep latency (the time that transpires between lights off and the onset of sleep), total sleep time, the time spent in each sleep stage, number of awakenings, WASO, and sleep efficiency (the ratio of total sleep time to time spent in bed, multiplied by 100). Normal sleep is separated into non-rapid eye movement (NREM) and rapid eye movement (REM) sleep. NREM sleep is further divided into 4 stages advancing from alert to asleep, with the lightest sleep occurring in Stage I sleep, and the deepest sleep occurring in Stage IV NREM sleep. NREM and REM sleep follow continuous cycles at approximately 90-minute intervals throughout the night. In addition, a diagnostic PSG includes channels to monitor air flow and limb movement to generate an AHI score and PLMAI score for evidence of sleep apnea and restless leg syndrome, respectively. A diagnostic PSG will be done on all patients on the first night of the placebo lead-in period. The diagnostic PSG will initially be scored locally by the site to determine if the patient still qualifies. The dPSG will be re-scored by the central facility. The rest of the PSGs will be done according to the schedule of evaluations (see Table 2) and scored by a central facility.

Digit Symbol Substitution Test (DSST)

The DSST is a subtest from the Wechsler Adult Intelligence Scale (Wechsler, 1955) that requires speedy recording of digits and symbols. It is used to measure psychomotor performance. It presents a code of 9 matched digits and symbols at the top of the test sheet. Patients are required to substitute a symbol for a digit from a code visible throughout the test. The number of correct items completed in 90 seconds is the score. Patients will be instructed on how to complete the DSST and take a practice test the first night of the placebo lead-in period. Additional DSST will be performed according to the schedule of evaluations (see Table 2) DSST will be administered approximately 1-hour after being awakened. The DSST will always be administered immediately after the VAS.

11.2. Subjective Assessments

- Pre- and post-sleep questionnaires

- VAS
- BWSQ

Pre- and post-sleep questionnaires

The pre- and post sleep questionnaires will be used in this study to assist assessing subjective sleep quality, depth of sleep, sleep latency, total sleep time, daytime function and daytime alertness. The pre-sleep questionnaire will be completed by the patient prior to study medication administration in the sleep clinic. The post-sleep questionnaire will be completed by the patient on both an inpatient and outpatient basis. In the sleep clinic, the PSQ will be completed approximately 1-hour after awakening immediately before the patients complete the VAS and DSST. A PSQ will also be given to each patient at the end of Visits 1 through 6 to take and complete at home. Patients should be instructed to complete the outpatient PSQ the morning of the next scheduled visit. These questionnaires will be completed by the patients as indicated on the schedule of evaluations (see Table 2).

Visual Analog Mood Scale (VAS)

The visual analog mood scale is a self-rated scale designed to assess feelings, affect, and mood, and their changes. The visual analog mood scale is a nonverbal measure of internal mood states (e.g., afraid, confused, sad, angry, energetic, tired, happy or tense). The scales have a neutral word at one end of a 100 mm line and a "mood" adjective at the other. The respondents indicate the point on the line that best describes how they are currently feeling. Patients will be instructed on how to complete the visual analog mood scale and will be given a practice test at Visit 2. Additional VAS will be performed according to the schedule of evaluations (see Table 2). VAS will be administered approximately 1-hour after being awakened immediately after the PSQ.

Benzodiazepine Withdrawal Symptom Questionnaire (BWSQ)

The Tyrer Benzodiazepine Withdrawal Symptom Questionnaire[13] is a self-reported questionnaire which records the main symptoms experienced during withdrawal from benzodiazepines in pharmacologically dependent patients. The questionnaire consists of 20 symptoms. Each symptom is rated from 0 to 2. The absence of a symptom is rated as 0. The presence of a symptom is rated as either 1 or 2, with 1 being moderate and 2 being severe. The maximum possible score is 40. There are two versions of the BWSQ. The first version (BWSQ-1) is a retrospective assessment of the most severe symptoms experienced at any time during the use of, or the withdrawal from benzodiazepines. The second version (BWSQ-2) assesses symptoms within the immediate past (2 weeks) and it is appropriate for evaluating the frequency of symptoms during withdrawal. This study will use BWSQ-2 which will be completed at Day 30 (± 2 days) and Day 37 (± 2 days) as part of the EOS assessments. The BWSQ will be given to patients before the battery of tests (approximately 1 hour after awakening).

12. ASSESSMENT OF SAFETY

12.1. Safety Parameters

Safety assessments should be conducted as specified in the Schedule of Evaluations (see Table 2). These assessments include: the regular monitoring and recording of all AEs and SAEs; the regular monitoring of hematology, blood chemistry and urinalysis values; the regular monitoring of vital signs; and the performance of physical examinations and electrocardiograms. Any amendments to this protocol that change the schedule of visits and procedures will be included in the clinical study report for this protocol.

12.1.1. Laboratory Evaluations

The Schedule of Evaluations (see Table 2) shows the time points at which blood will be collected for clinical laboratory tests and urine will be collected for urinalysis in the VP-VEC-162-3104 study.

Table 3 presents the clinical laboratory tests to be performed.

Table 3: Clinical Laboratory Tests

Category	Parameters
Hematology	Red blood cell count (RBC), hemoglobin, hematocrit, platelets, and white blood cell (WBC) count with differential (absolute counts of neutrophils, lymphocytes, monocytes, eosinophils, basophils)
Chemistry	
Electrolytes	sodium, potassium, chloride, magnesium
Liver function test	alkaline phosphatase, aspartate aminotransferase (AST [SGOT]), alanine aminotransferase (ALT [SGPT]), total bilirubin, gammaglutamyl transferase (GGT)
Renal function parameters	blood urea/blood urea nitrogen (BUN), creatinine
Other	glucose, calcium, albumin, total cholesterol, phosphorus, lactate dehydrogenase (LDH), total protein, uric acid, creatine kinase, amylase
Urinalysis	
Gross and chemical exam	color, appearance, specific gravity, pH, protein, glucose, ketone, blood, nitrite
Reflexive microscopic exam (will be done if any of the urinalysis testing is not negative)	RBC, WBC, epithelial cells, bacteria, casts, crystals

The clinical laboratory tests (hematology, chemistry, and urinalysis) will be performed by a certified central laboratory that will forward laboratory data to both the site and Vanda or its designee. Details regarding the collection, shipment of samples, reporting of results, and alerting of extreme values will be outlined in the Laboratory Manual, which will be supplied to all sites.

Values considered to be potentially clinically notable are provided in Appendix 21.1 for the Investigator's guidance. Any laboratory test result that the Investigator considers clinically significant will be repeated once to rule out laboratory error. For tests where a

persistent abnormality is considered to be drug related, repeat analyses will be performed until the cause is determined and either a return to normality occurs or the Investigator deems the abnormality to be of no clinical significance. Any laboratory test result that the investigator considers clinically significant must also be recorded as an adverse event.

12.1.2. Additional Laboratory Evaluations

Blood and urine samples will also be collected for the following evaluations: serum pregnancy, urine drug screen for drug abuse, and urine pregnancy test (over-the-counter pregnancy test). These assessments will be performed according to the Schedule of Evaluations (see Table 2).

12.1.3. Vital Signs and Body Measurements

Vital Signs

Vital signs will be assessed according to the Schedule of Evaluations (see Table 2).

Measurements will include the following:

- Body temperature (^{o}C)
- Respiratory rate
- Seated blood pressure (systolic and diastolic) –after being seated for approximately 5 minutes
- Seated pulse - after being seated for approximately 5 minutes

If the first vital sign value(s) is abnormal during Visit 1 (screening); the vital sign measurement(s) will need to be repeated. If the vital sign measurement(s) continue(s) to be abnormal (as defined in Section 7.2, Patient Exclusion Criteria), the patient will be excluded from the study.

After the patient signs the informed consent, vital sign values that the Investigator considers clinically significant will be recorded as adverse events for the patients that are enrolled into the study. Vital sign values considered to be potentially clinically notable are provided in Appendix 21.2 for the Investigator's guidance. The recording must be in the form of a clinical sign, symptom, or diagnosis, and not a mere description of the vital sign abnormality. Measurements will be repeated at medically appropriate intervals until they return to acceptable levels.

Body Measurements

Body measurements will be performed according to the Schedule of Evaluations (see Table 2).

- Body weight (kg) (at screening, Night 1, and EOS or ET)
- Height (cm) (at screening only)

12.1.4. Medical History and Physical Examinations

A full physical examination will be performed at screening and EOS or ET. An abbreviated physical examination will be conducted on Night 1 only if the patient reports a change in health or experiences an AE. Documentation of the physical examination will be included in the source documentation at the investigational site. Findings at screening will be recorded as medical history on the electronic CRF. Only changes from baseline physical examination findings that meet the definition of an adverse event will be recorded on the Adverse Events electronic CRF.

12.1.5. Electrocardiograms (ECGs)

A standard, resting 12-lead ECG will be obtained for each patient at the visits designated on the Schedule of Evaluations (see Table 2). A central facility will be used in this study for interpretation and analysis of ECGs. The time the ECG is performed will be recorded (using a 24-hr clock). Two original ECG tracings will be produced; one will be retained in the patient's clinical record, and the other will be kept for submission to the central ECG facility for reading as needed (in the event automated submission to central ECG facility can not be transmitted). Details concerning the ECG recording instructions will be provided to all Investigators in a separate manual prior to the start of the study.

To ensure accuracy in the ECG reading at baseline (Night 1), the ECG should be performed 3 times (about 5 minutes apart), whereas a single ECG reading is sufficient for other visits unless a clinically significant finding is present.

12.1.6. Pregnancy

Before enrolling a female of child-bearing potential in this clinical study, Investigators must review the following information with the patient:

- Informed consent requirements
- Risk of pregnancy
- Current contraceptive use
- Drug interactions with hormonal contraceptives
- Pregnancy prevention during the study

All females of child bearing potential, (defined as any female unless surgically sterile or postmenopausal at least 12 months) must be instructed to contact the Investigator immediately if they suspect they might be pregnant while participating in this study. Any pregnancy that occurs during study participation must be reported to Covance Drug Safety Services (Vanda's designee) using a Pregnancy Report and Paternal Exposure Form within 24 hours of learning of its occurrence and must be followed to determine outcome. If a patient becomes pregnant, she will be discontinued from the study.

Women using hormonal methods of birth control (e.g. oral contraceptives, patch and steroids) must use an additional method of birth control during the study and for one month after the last dose. I*n vitro* data suggests that VEC-162 could potentially induce the enzyme CYP3A4/5 which metabolizes hormonal contraceptives.

12.2. Definitions Related to Safety

12.2.1. Adverse Event

An ***adverse event*** (AE) is defined as any untoward medical occurrence in a clinical investigation patient that does not necessarily have casual relationship with treatment. An AE can therefore be any unfavorable and unintended sign (including clinically significant abnormal laboratory finding), symptom, or disease temporally associated with clinical study whether or not related to the investigational product.

Every attempt should be made to describe the AE in the form of a diagnosis. If a clear diagnosis has been made, individual signs and symptoms will not be recorded unless they represent atypical or extreme manifestations of the diagnosis, in which case they should be reported as separate events. If a clear diagnosis cannot be established, each sign and symptom must be reported individually.

12.2.2. Serious Adverse Event

Adverse events are classified as serious or non-serious. A ***serious adverse event*** is defined as any untoward medical occurrence that occurs during clinical study that meets one of the following criteria:

Table 4: SAE Criteria and Definitions

SAE Criteria	Definition
Death of Patient	An event that results in the death of the patient.
Life-Threatening	An event that, in the opinion of the Investigator, would have resulted in immediate fatality if medical intervention had not been taken. This does not include an event that would have been fatal if it had occurred in a more severe form.
Hospitalization	An event that results in an admission to the hospital for any length of time. This does not include an emergency room visit or admission to an outpatient facility.
Prolongation of Hospitalization	An event that occurs while the patient is hospitalized and prolongs the patient's hospital stay.
Congenital Anomaly/Birth Defect	An anomaly detected at or after birth, or any anomaly that results in fetal loss.
Persistent or Significant Disability/Incapacity	An event that results in a condition that substantially interferes with the activities of daily living of a study patient. Disability is not intended to include transient interruptions of daily activities or experiences of relatively minor medical significance such as headache, nausea, vomiting, diarrhea, influenza, and accidental trauma (e.g., sprained ankle).
Important Medical Event Requiring Medical or Surgical Intervention to	An important medical event that, based on medical judgment, may not be immediately life-threatening or result in death or hospitalization, but may jeopardize the patient and may require medical or surgical intervention to

SAE Criteria	Definition
Prevent Serious Outcome	prevent any of the outcomes listed above (i.e., death of a patient, life-threatening, inpatient hospitalization, prolongation of existing hospitalization, congenital anomaly, or persistent or significant disability/incapacity).

All AEs that do not meet the above criteria should be classified as ***non-serious adverse events.***

12.2.3. Adverse Event Follow-up

Patients with non-serious adverse events that are ongoing at the patient's last study visit must be followed until resolution or for 30 days after the patient's last study visit, whichever comes first. Non-serious adverse events that are reported during the 7 days following the patient's last study visit will be recorded on the Adverse Events electronic CRF and followed until resolution or for up to the 30 days after the patient's last study visit, whichever comes first. Serious adverse events will be followed until the event resolves or the event or sequelae stabilize. Serious adverse events that are reported within 30 days of the patient's last study visit should be reported as indicated in Section 12.5.

12.2.4. Adverse Event Reporting Period

Adverse events are to be recorded in the source documents from the time of the patient's informed consent signature until the end of the patient's study participation. Each adverse event, both serious and non-serious, will also be reported on the Adverse Events electronic CRF. CRF Completion Instructions will be provided to each investigational site. If the patient reports or the Investigator learns of a new AE(s) up to 7 days after the patient's last study visit or a new SAE(s) up to 30 days after the patient's last study visit, the investigational site personnel will ensure that these data are recorded on the Adverse Events electronic CRF for the study. The period during which an SAE must be reported may be extended if there is a strong suspicion that the event being reported is related to the study medication or a study procedure.

12.2.5. Pre-existing Condition

A preexisting condition is one that is present at the start of the study. A preexisting condition should be recorded as an adverse event if the frequency, intensity, or the characteristics of the condition worsen during the study period.

12.3. Relationship to Study Drug

Each AE is to be reported on the AE electronic CRF. The Investigator is responsible for making an assessment of the likelihood that an AE is causally related to the study medication. The Investigator should choose one of the five choices of causality.

- **Certain**: occurs in a reasonable time after study drug administration and cannot be explained by concurrent disease or drugs. The event should

respond to withdrawal of study drug (de-challenge) and recur with re-challenge when clinically plausible.

- **Probable:** occurs in a reasonable time after study drug administration and it is unlikely to be to be attributed to concurrent disease or drugs, and it has a response to de-challenge. Re-challenge information is not required to fulfill this definition.
- **Possible:** occurs in a reasonable time after study drug administration, but could be related to concurrent disease or drugs. De-challenge information may be lacking or unclear.
- **Unrelated:** the event has an improbable temporal relationship (too soon, or too late after study drug, or study drug is not taken) and is plausibly related to other drugs or underlying disease.
- **Unassessable:** available information is insufficient, contradictory, and cannot be supplemented or verified at the time of the report. **This assessment will be considered as "related" for all expedited reports until an alternative assessment is made.**

12.4. Recording Adverse Events

12.4.1. Adverse Events during Study Period

At each study visit, the Investigator must seek information on adverse events by questioning the patient and, as appropriate, by examining the patient. Information on all adverse events should be recorded immediately in the source document, and also in the appropriate adverse event module of the electronic CRF. All signs, symptoms, and abnormal diagnostic procedure results that are considered clearly related should be grouped and recorded in the source document as one diagnosis. All adverse events occurring during the study period must be recorded.

12.4.2. Post-study Adverse Event

At the last scheduled visit, the Investigator should instruct each patient to report any subsequent event(s) that the patient or the patient's personal physician believes might reasonably be related to participation in this study. The Investigator should notify the study sponsor of any death or adverse event occurring at any time after a patient has discontinued or terminated study participation that may reasonably be related to this study. The Investigator should also notify the sponsor if they become aware of the development of cancer or a congenital anomaly in any offspring conceived subsequent to a patient's study participation.

12.4.3. Abnormal Laboratory Values

Abnormal laboratory values or test results should not generally be considered adverse events, unless deemed clinically significant by the investigator. Assessment of signs or symptoms or requirement for therapeutic intervention should be considered when

determining clinical significance. Record clinical significant laboratory values on the Adverse Events Case Report Form using an appropriate diagnostic description.

12.5. Reporting Adverse Events

12.5.1. Study Sponsor Notification by Investigator

All serious AEs should be reported by fax within 24 hours to the Vanda Drug Safety designee (Covance Drug Safety Services) using the Serious Adverse Event Report form. The Investigator will keep a copy of the SAE Report form on file at the study site. The SAE form and detailed instructions are provided in the Study Manual.

The initial report should contain as much information about the event as possible. Follow-up reports will provide any information missing at the time of the initial report (e.g., an end date for the adverse event or laboratory values received after the report). In addition, significant new information on ongoing SAEs should be provided promptly to Covance Drug Safety Services.

The initial and follow-up reports should identify patients by a unique patient ID rather than by patients' names, personal identification numbers, and/or address.

At the time of the initial report, the following information listed in Table 5 should be provided at a minimum.

Table 5: SAE Reporting Information

• Protocol Number<br>• Site Number<br>• Patient ID number<br>• Patient Initials<br>• A description of the event<br>• Date of onset<br>• Current status	• Intensity level of SAE<br>• Action taken regarding study medication administration<br>• The reason the event is classified as serious<br>• Investigator assessment of study medication relationship and SAE<br>• If the blind was broken

12.5.2. EC/IRB Notification by Investigator

Reports of all SAEs (including follow-up information) must be submitted by the Investigator to the EC/IRB within 10 working days. Copies of each report and documentation of EC/IRB notification and receipt will be kept in the Clinical Investigator's Study File.

12.6. Unblinding Procedures

The patients and the medical staff will not be aware of which treatment is being administered. Data will remain blinded until clinical data have been reviewed, at which time the randomization schedule may be released for the purposes of assessment of drug safety.

Only if a patient's medical condition warrants, such as a medical emergency for which treatment requires knowledge of what study medication was given, may the Investigator break the blind to determine if the patient received active drug or placebo. In most instances of medical emergency, prior approval to break the code must be granted by the Medical Monitor. However, the code can be broken on the Investigator's request (by telephoning the IVRS) in those cases where the patient's condition is so severe that time would not permit prior approval. In any case, the study monitor must be informed as soon as possible by telephone following the event and by letter or e-mail explaining the details of the case with accompanying diagnostic reports, where appropriate. The Investigator must ensure that any communication with the Medical Monitor is blinded even after the Investigator has broken the blind.

13. PHARMACOGENOMIC ASSESSMENT

Patients will be offered the option of participating in an additional study to identify genetic factors that may predict response to treatment with VEC-162 and/or relative susceptibility to side effects. Participating patients must provide a separate written informed consent to permit the collection of a blood sample for pharmacogenomic analysis. The pharmacogenomic protocol is described in Appendix 21.3. While this exploratory assessment is optional to the patient, it should be noted that *PER3* genotyping is mandatory. Once this genotyping is finalized, any remaining sample will be destroyed by the sponsor and/or the sponsor's representative.

14. STATISTICS

14.1. Statistical Methods

This section describes the planned statistical analyses in general terms. A complete description of the methodology will be specified in a statistical analysis plan (SAP), which will be finalized prior to database lock. Any changes in the statistical methods described in this protocol that occur prior to database lock will be documented in the statistical analysis plan and will not require a protocol amendment.

14.1.1. General

The primary efficacy variable is the average Latency to Persistent Sleep (LPS) computed via PSG assessments from nights 1 and 8. The primary null hypothesis to be tested is that there are no differences among the treatments.

Statistical analyses will be performed using two-sided test. A 0.05 significance level will be used in all tests of treatment differences. Test for interaction will utilize a 0.10 statistical significance level.

For the primary efficacy endpoint, the type I error will be controlled via Fisher's protected least significant difference (LSD). Using this procedure, an overall test of the treatment effect will be conducted and pairwise comparisons will be examined only if the overall treatment is significant at the 0.05 alpha level. Pairwise comparisons will also be examined at the 0.05 significance level.

In addition, for labeling purposes only, the experiment wise Type I error will be controlled to a maximum of 5%. A gatekeeper strategy will be employed such that any secondary endpoints will be considered for statistical significance only if the primary endpoint to be statistically significant.

Data will be summarized by treatment group (and by visit when applicable), with respect to demographic and baseline characteristics, efficacy variables, and safety variables.

Summary statistics will include the mean, N, standard deviation, median, minimum, and maximum values for continuous variables, and frequencies and percentages for categorical variables.

Continuous variables such as latency to persistent sleep (LPS), wake after sleep onset (WASO), total sleep time (TST) and sleep efficiency (SE) will be analyzed using an ANCOVA model with treatment and pooled center as main effects, baseline as a covariate and the treatment-by-baseline interaction. An exploratory analysis will be performed to explore whether there are treatment-by-center interactions. If a treatment-by-center interaction is detected, the interaction will be explored in an ad-hoc manner.

Categorical variables will be analyzed using Cochran-Mantel-Haenszel (CMH) test blocking on centers (or pooled centers).

Details of the model and the analyses will be specified in the SAP. All statistical analyses will be performed using SAS®, Version 8.2 or higher.

14.2. Sample Size and Accrual

The sample size is based on the average LPS computed via PSG assessments from nights 1 and 8. Based on a t-test to detect a 15 minute difference between VEC-162 treated patients and placebo patients, and assuming a common standard deviation of 30 minutes, a sample size of 86 patients per treatment arm is needed in order to have 90% power with a two-sided alpha of 0.05. The standard deviation is based on data found in literature. Considering a 1:1:1 randomization between VEC-162 20mg, VEC-162 50mg and placebo, and a 20% drop-out rate, a total of 324 patients (108 per arm) will be needed.

A re-estimation of the sample size will be done through an adjusted one-sample variance procedure. In this procedure, a blinded variance estimation is obtained by adjusting the one-sample variance of the pooled sample.

Let N and VAR be the respective preliminary sample size and variance originally estimated (as per above). Let N(s) and VAR(s) be the respective sample size and variance of the pooled blinded sample at this sample-size re-assessment look. Then according to Zucker et al. (13), the following estimation of the variance (VAR(adj)) will be used to generate an adjusted sample size N(adj);

$$VAR(adj) = VAR(s) - N(s)\, D^2 / 2(2N(s)-1),$$

where D is equal to a 15 point change from baseline

N(adj) will then be generated using a two-group t-test procedure with a 0.05 two-sided significance level and the variance VAR(adj).

Since this sample size re-assessment is not intended to stop the trial early due to efficacy but only to provide a variance re-estimation and a sample size recalculation, then after the recruitment of the first n(s) patients,

- if N(adj) <= N, then a further N-N(s) patients will have to be recruited until the initially planned sample size N is reached.
- Otherwise if N(adj) > N, then a further N(adj)-N(s) patients will have to be recruited.

As the procedure does not require unblinding of the treatment code during the ongoing trial nor produce any estimation of a treatment effect hypothesis testing, then no adjustment to control the nominal significance level is required.

The required sample size could also be adjusted if the drop-out rate differs from the estimated drop-out rate. This change would be made via a protocol amendment.

14.3. Interim analysis

No interim analyses are planned.

14.4. Patient Populations for Analysis

The following analysis populations will be defined for this study:

Randomized: will include any patient randomized into the study. This population can be referred to as the Intent-to-Treat population.

Full Analysis: will include any patient randomized into the study that receive a dose of study drug and have post-baseline polysomnography data. This analysis population may also be referred to as the modified Intent-To-Treat population.

Safety: Any patient randomized into the study that receives a dose of study drug.

Per-Protocol: Any patient who is randomized and receives the protocol required study drug exposure and required protocol processing, e.g., patients who undergo all five overnight visits [Nights 1, 8, 22, 29 (± 2 days) and 36] in which PSG is assessed.

Primary analyses of efficacy endpoints (primary and secondary) will be performed on the Full Analysis Set. Additionally, these analyses will be repeated on the Per-Protocol set. Safety summaries will be based on Safety set. Patient characteristics will be presented for all patients randomized.

14.5. Pooling of Centers

Low enrolling sites will be pooled for analysis and the pooling algorithm will be determined prior to breaking the blind. The goal of pooling low enrolling sites is to have a sufficient number of patients per treatment group within site for the analysis models and for the evaluation of the treatment-by-site interaction for the primary endpoint. Unless otherwise specified, the pooled sites will only be used in the analyses where site is an effect. The actual sites rather than the pooled sites will be specified in data listings.

14.6. Demography and Other Baseline Data

Demographic data and patient characteristics at screening/baseline will be listed and summarized by treatment group for all randomized patients using descriptive statistics.

Analysis will be conducted to determine differences of the treatment groups in the demographic characteristics. Baseline comparability will be evaluated based on the pooled data from all centers. To determine comparability of the treatment groups at baseline, continuous demographic and clinical variables will be analyzed by an ANOVA model that includes a main effect for treatment, those that are ordinal by the Mantel-Haenszel test, and those that are dichotomous by the chi-square test or Fisher’s exact test. If there is treatment group differences at the 0.10 level of significance in demographic or baseline clinical variables, these variables may be added as stratification variables or covariates to the efficacy analyses (will be considered as exploratory sensitivity analyses only).

Medical history (medical conditions ending prior to start of study medication) will be summarized treatment group using the system organ class (SOC). Refer to section 13.10.1 for details

Deviations from inclusion/exclusion criteria as well as patient's status will only be listed.

14.7. Study Medication

The number of patients at each study visit will be summarized by treatment group.

The compliance to study medication, as recorded in the CRF will also be summarized by treatment group.

14.8. Concomitant Therapy

Concomitant medications (medications present while on study medication) will be recorded throughout the study and at early termination. These medications will be coded using the WHO-drug dictionary. The number of patients from the Safety sample using prior or concomitant medications will be categorized by the WHO-drug category and preferred term, and presented for each treatment group. In any given category (e.g., drug category) a patient will be counted only once.

14.9. Efficacy Data

14.9.1. Efficacy Outcomes

The efficacy outcomes consist of the following objective assessments:

- Polysomnography (PSG) including: LPS, WASO, TST and SE
- Digit Symbol Substitution Test (DSST)

And the following subjective assessments:

- Pre- and post-sleep questionnaires (Pre-SQ and PSQ)
- Visual Analog Mood Scale (VAS)
- Benzodiazepine Withdrawal Symptom Questionnaire (BWSQ)

14.9.1.1. Primary Efficacy Outcome

The primary efficacy variable is the average latency to persistent sleep (LPS) as measured by polysomnography (PSG) on Nights 1 and 8.

14.9.1.2. Secondary Efficacy Outcomes

The secondary efficacy variables are:

- the average WASO, TST and SE as measured by PSG on Nights 1 and 8.
- the average LPS, WASO, TST and SE as measured by PSG on Nights 22 and 29
- the LPS, WASO, TST and SE as measured by PSG on Nights 1, 8, 22 and 29
- rebound effects through the LPS, WASO, TST and SE as measured by PSG on Night 36
- subjective assessments such as: sleep quality, depth of sleep, sleep latency (sSL), total sleep time (sTST), daytime function and daytime alertness
- residual effects through DSST score and VAS score
- subjective withdrawal symptoms after 5 weeks of VEC-162 treatment as measured by the benzodiapine withdrawal symptom questionnaire (BWSQ)
- contribution of a PER3 gene polymorphism on the primary and secondary efficacy endpoints.

14.9.2. Efficacy Analysis

Continuous variables will be analyzed parametrically using ANOVA or ANCOVA models, and categorical variables will be analyzed using the generalized Cochran-Mantel-Haenszel (CMH) test with (pooled) site as a stratum.

14.9.2.1. General Data Analysis Considerations

Imputation of missing data

For the average PSG parameter values calculated as an average over 2 nights (Night 1 and 8 or Nights 22 and 29), if one of the 2 nights entering in the calculation of the average value is missing, then it will be imputed using the non-missing night value, e.g. if Night 1 is missing then it will be imputed using the Night 8 value and if Night 8 is missing, it will be imputed using the Night 1 value. If both post-baseline values are missing then the average value for this PSG parameter will be imputed using the baseline value only (Baseline Observation Carried Forward - BOCF).

The observed data will otherwise be used in the analyses.

14.9.2.2. Statement of the Null and Alternate Hypotheses

Statistical analyses will be performed using two-sided test. A 0.05 significance level will be used in all tests of treatment differences. Test for interaction will utilize a 0.10 statistical significance level.

The type I error will be controlled via Fisher's protected least significant difference (LSD). Using this procedure, an overall test of the overall treatment effect will be conducted and pairwise comparisons will be examined only if the overall treatment is significant at the 0.05 alpha level. Pairwise comparisons will also be examined at the 0.05 significance level.

14.9.2.3. Analysis of the Primary Endpoint

The primary efficacy variable, i.e., average LPS value over Nights 1 and 8, will be analyzed using an ANCOVA model that includes main effects for treatment and (pooled) site, the baseline assessment as a covariate and the treatment-by-baseline interaction term to check the consistency of treatment differences across various baseline score levels.

If the data for the primary efficacy variable does not satisfy underlying assumptions of normality and homogeneity of variance, then a data transformation such as a Log transformation (base 10) or a Logit may be applied, or a non-parametric model may be used to analyze the data.

14.9.2.4. Analysis of the Secondary Endpoints

The continuous secondary efficacy variables will be analyzed using a similar parametric model as the one used in the analysis of the primary efficacy variable, i.e. an ANCOVA model that includes main effects for treatment and (pooled) site, the baseline assessment as a covariate and the treatment-by-baseline interaction term to check the consistency of treatment differences across various baseline score levels.

Categorical secondary efficacy parameters will be analyzed using a CMH test stratifying by (pooled) site.

14.9.2.5. Exploratory Efficacy Analyses

Sensitivity analyses on the primary efficacy variable may be conducted using the alternate imputation methods such Baseline Observation Carried Forward (BOCF) or Mixed-Model Repeated Measures (MMRM) imputed data. The data will then be analyzed using the same ANCOVA model that was used to analyze the primary efficacy variable. This will be done for the ITT population (Full analysis) only.

Adding the treatment-by-site (pooled site) interaction term to the ANCOVA model that was used in the analysis of the primary efficacy variable. If significant at the 0.10 level, summary and inferential statistics will be presented for each (pooled) site. This will be done for the ITT population (Full analysis) only.

Subgroup analyses on selected efficacy variables may be conducted using the ITT (Full analysis) population if deemed necessary.

14.9.2.6. Graphs of Efficacy

Graphical representation (over time) for the primary efficacy parameter and other efficacy parameters of interest will be depicted.

14.10. Safety Data

The primary assessment of safety will be based on the frequency of treatment-emergent adverse events and on the frequency of clinically notable abnormal vital signs and laboratory values.

14.10.1. Adverse Events

Adverse events will be recorded throughout the study and at early termination. Adverse events and medical conditions will be coded using the Medical Dictionary for Regulatory Activities (MedDRA) coding dictionary. Treatment-emergent adverse events will be defined as those events, which are newly occurring or worsening from baseline. In all cases only treatment emergent adverse events will be summarized.

Treatment-emergent adverse events will be summarized by treatment group, by presenting, for each treatment group, the number and percentage of patients having any treatment-emergent AE, having an AE in each body system, and having each individual AE. (Note: In any given category [e.g. body system] a patient will only be counted once.) Similar displays will be provided for prior (conditions ending prior to the first dose of study medication) and current conditions present while on study medication) medical conditions.

Adverse events will further be categorized by severity, relationship to study medication, and action taken. Other information collected will be listed, as appropriate. Any event starting more than 3 days after the final dose of study medication will be excluded from the above tables and only listed, unless the event caused discontinuation.

14.10.2. Laboratory Data

Laboratory data will be summarized for each treatment group by presenting the proportions of patients with clinically notable abnormalities (Appendix 21.1) as defined below; shift tables, baseline to most extreme post-baseline value, using normal ranges (extended normal ranges for laboratory data; ±10% of normal range for electrolytes [sodium, potassium, and calcium] and ±15% of the normal range for all other measures); summary statistics of raw data and change from baseline values (means, medians, standard deviations, ranges).

Clinically notable values will be identified according to the criteria identified in the FDA's "Supplementary Suggestions for Preparing an Integrated Summary of Safety Information in an Original NDA Submission and for Organizing Information in Periodic Safety Updates" (Revised 2-APR-87) provided by the FDA Division of Neuropharmacological Drug Products (DNDP).

14.10.3. Vital Signs and Body Measurements

Data from vital signs and body measurements will be listed, clinically notable values as previously defined (Appendix 21.2) will be flagged, and any other information collected will be listed. Data will be summarized by treatment group using mean change from baseline and proportions of patients with values outside the normal range, and values that were clinically notable.

14.10.4. Electrocardiogram (ECG)

Results from the ECG will be listed for each patient. These data will also be summarized for each treatment group by presenting patients with newly occurring or worsening ECG abnormalities (as defined by Vanda designated central lab facility); shift tables, baseline to most extreme post-baseline value; summary statistics of raw data and change from baseline values (means, medians, standard deviations, ranges).

Whenever multiple ECG readings are done on the same day, the average of these readings will be taken for the data summaries.

15. DIRECT ACCESS TO SOURCE DOCUMENTS

15.1. Definition of Source Document

Source data is all information, original records of clinical findings, observations, or other activities in a clinical trial necessary for the reconstruction and evaluation of the trial. Source data are contained in source documents. Examples of these original documents and data records (ICH E6, Section 6.10) include, but are not limited to: hospital records, clinical and office charts, laboratory notes, memoranda, patients' diaries or evaluation checklists, pharmacy dispensing records, recorded data from automated instruments, copies or transcriptions certified after verification as being accurate and complete, microfiches, photographic negatives, microfilm or magnetic media, X-rays, patient files, and records kept at the pharmacy, at the laboratories, and at medico-technical departments involved in the clinical trial. The investigator is required to maintain adequate and accurate source documents that record all observations and other data pertinent to this study.

15.2. Study Monitoring

The Sponsor's or CRO's monitor will maintain contact with the Investigator and designated staff by telephone, and/or letter, and/or email between study visits. Monitoring visits to each investigational site will be conducted by the assigned monitor as frequently as stipulated in the monitoring plan. The Investigator must designate an adequate space to conduct the monitoring visit and will allocate adequate time for Vanda's monitoring activities. The electronic CRFs and patient's corresponding original medical records (source documents) are to be fully available for review by the monitor.

The purposes of clinical trial monitoring are to verify that:

- the rights and well-being of the human patients are protected;
- the reported trial data are accurate, complete and verifiable from source documents;
- the conduct of the trial is in compliance with the currently approved protocol/amendment(s), with GCP, and with applicable regulatory requirement(s).

15.3. Audits and Inspections

In addition to routine monitoring procedures, Vanda may request an independent Good Clinical Practice Quality Assurance contractor to perform audits of clinical research activities to evaluate compliance with principles of Good Clinical Practice. The Investigator will ensure that the compliance or quality assurance reviewer is allowed to:

- review all of the study-related documents (e.g., study records and source documents) and study related facilities (e.g., pharmacy, diagnostic laboratory)
- discuss the conduct of the study with the Investigator

A regulatory authority may also wish to conduct an inspection (during the study or even after its completion). If an inspection is requested by a regulatory authority, the Investigator must inform the CRO and Vanda immediately that this request has been made, and promptly forward copies of the audit reports to Vanda.

16. QUALITY CONTROL AND QUALITY ASSURANCE

This study will be organized, performed, and reported in compliance with the protocol, Standard Operating Procedures (SOPs), working practice documents, and applicable regulations and guidelines. Site visit audits will be made periodically by the Sponsor's (or CRO's) qualified compliance auditing team, which is an independent function from the study conduct team.

16.1. Data Collection

The study CRF is the primary data collection instrument for the study. For this study, the CRF will be completed electronically. All data requested on the electronic CRF must be recorded. All missing data must be explained. A detailed completion of the CRF guideline manual will be included in the Investigator's manual. Monitors will highlight any discrepancies found in the documentation of study conduct and ensure that appropriate site personnel address the discrepancies.

16.2. Clinical Data Management

Data from the electronic CRFs and other external data (e.g. laboratory data) will be entered into a clinical database as specified in the Sponsor [or CRO's] data management plan. Quality control and data validation procedures will be applied to ensure the validity and accuracy of the clinical database.

16.3. Database Quality Assurance

The clinical database will be reviewed and checked for omissions, apparent errors, and values requiring further clarification using computerized and manual procedures. Data queries requiring clarification will be documented and returned to the investigational site for resolution. Only authorized personnel will make corrections to the clinical database, and all corrections will be documented in an audit trail. A quality assurance audit will be performed prior to database lock.

17. ETHICS

17.1. Ethics Review

This protocol and any amendments will be submitted to a properly constituted EC or IRB, in agreement with local legal prescriptions (ICH 3.1-3.4), for formal approval of the study conduct. In accordance with ICH 4.4, the decision of the IRB concerning the conduct of the study will be made in writing to the Investigator and a copy of this decision will be provided to the sponsor before commencement of this study and during the duration of the study (as needed). The Investigator should provide a list of EC/IRB members and their affiliation to the sponsor

All patients for this study will be provided a consent form describing this study and providing sufficient information for patients to make an informed decision about their participation in this study. This consent form will be submitted with the protocol for review and approval by the EC/IRB for the study. The formal consent of a patient by the investigative site, using the EC/IRB-approved consent form, must be obtained before that patient is submitted to any study procedure. This consent form must be signed by the patient, or patient's legally acceptable surrogate, and the Investigator-designated research professional obtaining the consent.

17.2. Ethical Conduct of the Study

This study is to be conducted according to US and international standards of Good Clinical Practice, as described in the following documents:

1. ICH Harmonized Tripartite Guidelines for Good Clinical Practice 1996.
2. Directive 91/507/EEC, The Rules Governing Medicinal Products in the European Community.
3. US Code of Federal Regulations (CFR) dealing with clinical studies (21 CFR including parts 50 and 56 concerning informed consent and IRB regulations).
4. Declaration of Helsinki, concerning medical research in humans (Recommendations Guiding Physicians in Biomedical Research Involving Human Subjects, Helsinki 1964, amended Tokyo 1975, Venice 1983, Hong Kong 1989, and Edinburgh 2000).

The Investigator agrees, when signing the protocol, to adhere to the instructions and procedures described in it and thereby to adhere to the principles of Good Clinical Practice.

17.3. Written Informed Consent

Informed consent will be sought by the Investigator or Investigator-designated research professional at each site from each potential participant in a private, neutral setting. The

informed consents will include all required components established by the Department of Health and Human Services (DHHS) (45 CFR 46.116) and Food and Drug Administration (FDA) regulations (21 CFR 50.20). The informed consent forms will be approved by the IRB prior to seeking consent from any potential participant from each site. Potential participants will have sufficient time to consider their participation in the study and the opportunity to discuss their questions or concerns with the Investigator, family, friends, or others (if desired) before signing. The consent forms will be reviewed with the participant (or the participant's representative), and signed and dated by the participant or the participant's representative before any research procedures (including screening) or research data collection begin. Patient will receive a copy of the informed consent documents. Informed consents obtained from each participant will be documented in both the source documents and the electronic CRF. Patients will have the possibility to withdraw from the study at anytime.

An additional informed consent regarding the optional pharmacogenomic assessments will also be given to the potential participant. The pharmacogenomic informed consent form will also be submitted for ethical approval. If a patient chooses not to participate in the pharmacogenomic assessment, the patient’s ability to participate in the main research study will not be affected in any way.

18. DATA HANDLING AND RECORD KEEPING

To maintain patient confidentiality, all evaluation forms, reports, and other records that leave the site will be identified only by the Patients ID and patient initials. All records will be kept in a secure area with limited access. All computer entry and networking programs will be performed using Patient ID only. Clinical information will not be released without prior written permission of the patient (as indicated in the informed consent), except as necessary for monitoring by the IRB, the FDA, or other regulatory agencies and the sponsor.

Information about study patients will be managed according to the requirements of the Health Insurance Portability and Accountability Act of 1996 (HIPAA). Those regulations require a signed patient authorization informing the patient of the following:

- What protected health information (PHI) will be collected from patients in this study
- Who will have access to that information and why
- Who will use or disclose that information
- The rights of research patients to revoke their authorization for use of their PHI.

In the event that a patient revokes authorization to collect or use PHI, the Investigator, by regulation, retains the ability to use all information collected prior to the revocation of patient authorization. For patients who have revoked authorization to collect or use PHI, attempts should be made to obtain permission to collect at least vital data (i.e., that the patient is alive) at the end of their scheduled study period. Any identifiable sample that has already been collected will be destroyed.

18.1. Retention of Records

It is the Investigator's responsibility to retain essential study documents for at least 2 years after the last approval of a marketing application in his/her country for the investigational product and until there are no pending or contemplated marketing applications in his/her country, or at least 2 years have elapsed since the formal discontinuation of clinical development of the investigational product. These documents should be retained for a longer period if required by an agreement with the sponsor. In such an instance, it is the responsibility of the sponsor to inform the Investigator/institution as to when these documents no longer need to be retained. The Investigator must contact Vanda prior to destroying any records associated with the study.

19. ADMINISTRATIVE PROCEDURES

19.1. Changes to the protocol

Except for a change that is intended to eliminate an immediate hazard to patients, the approved protocol shall be conducted as described. Any significant protocol deviation must be documented in the source documents.

Any change or addition to this protocol requires a written protocol amendment that must be approved by Vanda and the Investigator before implementation. Amendments significantly affecting the safety of patients, the scope of the investigation or the scientific quality of the study require additional approval by the IRB. A copy of the written approval of the IRB, which becomes part of the protocol, must be given to the Vanda monitor. In addition, amendments that affect the informed consent require a revised and IRB-approved informed consent, before changes in study procedures are implemented. These requirements for approval should in no way prevent any immediate action from being taken by the Investigator or by Vanda in the interests of preserving the safety of all patients included in the study. If an immediate change to the protocol is felt to be necessary by the Investigator and is implemented by him/her for safety reasons, the Vanda monitor should be notified, and the IRB/EC at the center should be informed within 10 working days.

Amendments affecting only administrative aspects of the study do not require formal protocol amendments or IRB approval but the IRB must be kept informed of such administrative changes.

19.2. Periodic Reports to IRB/EC

The Investigator must provide reports of the progress, or completion, termination or discontinuation of the study to the IRB(s)/EC(s) at appropriate intervals: at least yearly for all Investigational New Drug Application (IND) studies or more frequently if required by applicable regulations and guidelines, or institution procedures.

19.3. Discontinuation of Study

Vanda reserves the right to discontinue any study for administrative reasons at any time. If appropriate, reimbursement for reasonable expenses will be made.

19.4. Publication of Results

Manuscript(s) for publication, texts of presentations, abstracts of papers, and similar material should be submitted to Vanda for review and comment at least sixty (60) days prior to submission for publication, public dissemination or review by a program committee. Upon request from Vanda, the site shall remove any confidential information (other than study results) prior to submitting or presenting the materials. Vanda shall

notify the Investigator in writing within sixty (60) days of receipt of such draft whether it contains information deemed to be confidential or information that if published within thirty (30) days would have an adverse effect on Sponsor's business interests, including but not limited to a patent application in which Vanda owns an interest. In the latter case, Vanda may request a delay and Investigator agrees to delay publication or presentation for a period not exceeding ninety (90) days. An independent joint publication may be authored by investigators from the multiple sites. The Investigator, therefore, agrees not to independently publish the results of the study before the publication of such multi-site paper, if applicable. No party heretofore mentioned shall use any other party's name, or Vanda's name, in connection with any advertising, publication, or promotion without prior written permission.

19.5. Investigator Agreement

This protocol is being provided to me for conducting a clinical trial for Vanda. The information contained in the protocol is confidential and proprietary to Vanda. Study documents provided by Vanda (protocols, Investigator's Brochures, and other material) will be stored appropriately to ensure their confidentiality.

I may disclose the contents of this protocol to study personnel under my supervision and my Institutional Review Board for the purpose of conducting this trial only.

The information provided by Vanda to me may not be disclosed to others without direct written authorization from Vanda, except to the extent necessary to obtain informed consent from subjects who wish to participate in the trial.

I have read the protocol and agree that it contains all necessary details for carrying out the study as described. I will conduct this protocol as outlined and according to the moral, ethical, and scientific principles governing the Declaration of Helsinki and the principles of GCP as described in 21 CFR parts 50, 54, 56, and 312 and applicable local requirements.

I understand that should the decision be made by Vanda to terminate prematurely or suspend the clinical trial at any time, with or without cause, such decision will be communicated to me in writing. Conversely, if I decide to withdraw from execution of the clinical trial, I will immediately communicate such decision in writing to Vanda.

Investigator

Name: ____

Title: ____

Signature: ______________________________________

Date: ____

Protocol Number: VP-VEC-162-3104

Site: ____

Address: ____

____

Telephone: ____

Note: Add name, title, address, and telephone number of the qualified physician who is responsible for trial site-related medical decisions if someone other than the Investigator.

20. REFERENCES

Reference List

(1) Colten HR, Altevogt B.M. Sleep Disorders and Sleep Deprivation: An Unmet Public Health Problem. The National Academies Press, 6 A.D.

(2) Roth T. Prevalence, associated risks, and treatment patterns of insomnia. J Clin Psychiatry 2005; 66 Suppl 9(10-3):42-43.

(3) Roth T, Roehrs T. Insomnia: epidemiology, characteristics, and consequences. Clin Cornerstone 2003; 5(3):5-15.

(4) Eddy M., Wallace JI. Insomnia. Am Fam Physician 1999; 59(7):1911-1916.

(5) Morin AK. Strategies for Treating Chronic Insomnia. Am J Manag Care 2006; 12:S230-S245.

(6) Sanger D.J. The search for novel antipsychotics: pharmacological and molecular targets. CNS Drugs 2004; 18(Suppl 1):9-15.

(7) Terzano M.G., Rossi M., Palomba V., Smerieri A, Parrino L. New drugs for insomnia: comparative tolerability of zopiclone, zolpidem and zaleplon. Drug Saf 2003; 26(4):261-282.

(8) Drover D.R. Comparative pharmacokinetics and pharmacodynamics of short-acting hypnosedatives: zaleplon, zolpidem and zopiclone. Clin Pharmacokinet 2004; 43(4):227-238.

(9) Patat A., Paty I., Hindmarch I. Pharmacodynamic profile of Zaleplon, a new non-benzodiazepine hypnotic agent. Hum Psychopharmacol 2001; 16(5):369-392.

(10) Van Den Heuvel CJ, Kennaway D.J., Dawson D. Thermoregulatory and soporific effects of very low dose melatonin injection. Am J Physiol 1999; 276(2 Pt 1):E249-254.

(11) Cajochen C, Krauchi K, Wirz-Justice A. The acute soporific action of daytime melatonin administration: effects on the EEG during wakefulness and subjective alertness. J Biol Rhythms 1997; 12(6):636-643.

(12) Dawson D., Gibbon S., Singh P. The hypothermic effect of melatonin on core body temperature: is more better? J Pineal Res 1996; 20(4):192-197.

(13) Tyrer P, Murphy S, Riley P. The Benzodiazepine Withdrawal Symptom Questionnaire. J Affect Disord 1990; 19(1):53-61.

(14) Zucker DM, Wittes JT, Schabenberger O, Brittain E. Internal pilot studies II: Comparison of various procedures. Statistics in Medecine 1999; 18:3493-3509.

21. APPENDICES

21.1. Laboratory Ranges Used to Identified Clinically Notable Abnormal Laboratory Values

Criteria for identifying laboratory values as Potentially Clinically Notable Abnormalities are based on the Guidelines for the Division of Neuropharmacological Drug Products, US Food and Drug Administration (revised on April 2, 1987).

Variable		Criterion Values	
		Standard Units	SI Units
**Chemistry**			
SGOT		≥ 3 x Upper Limit Normal	
SGPT		≥ 3 x Upper Limit Normal	
Alkaline Phosphatase		≥ 3 x Upper Limit Normal	
LDH		≥ 3 x Upper Limit Normal	
BUN		≥ 30 mg/dL	≥ 10.7 μM
Creatinine		≥ 2.0 mg/dL	≥ 176.8 μM
Uric Acid	Male	≥ 10.5 mg/dL	≥ 624.6 μM
	Female	≥ 8.5 mg/dL	≥ 505.6 μM
Bilirubin (Total)		≥ 2.0 mg/dL	≥ 34.2 μM
**Hematology**			
Hematocrit	Male	≤ 37%	
	Female	≤ 32%	
Hemoglobin	Male	≤ 11.5 g/dL	
	Female	≤ 9.5 g/dL	
Platelets		≤ 75,000/mm^3 or ≥	≤ 75 x 10^9/L or ≥ 700 x 10^9/L
Leukocytes		≤ 2,800/mm^3 or ≥ 16,000/mm^3	≤ 2.8 x 10^9/L or ≥ 16 x 10^9/L
Eosinophils		≥ 10%	
Neutrophils		≤ 15%	
**Urinalysis**			
Protein		Increase of ≥ 2 units	
Glucose		Increase of ≥ 2 units	
Casts		Increase of ≥ 2 units	

21.2. Vital Signs Values

Criteria for identifying vital signs values as Potentially Clinically Notable Abnormalities are based on the Guidelines for the Division of Neuropharmacological Drug Products, US Food and Drug Administration (revised on April 2, 1987).

Variable	Criteria		Change Relative to Baseline
Heart Rate	≥ 120 bpm	and an	increased of ≥ 15 bpm
	≤ 50 bpm	and a	decrease of ≥ 15 bpm
Systolic Blood Pressure	≥ 180 mmHg	and an	increase of ≥ 20 mmHg
	≤ 90 mmHg	and a	decrease of ≥ 20 mmHg
Diastolic Blood Pressure	≥ 105 mmHg	and an	increase of ≥ 15 mmHg
	≤ 50 mmHg	and a	decrease of ≥ 15 mmHg
Temperature	≥ 38.3 o C	and a	change of ≥ 1.1 o C
	≥ 101o F	and a	change of ≥ 2o F
Weight	--		change of ≥ 7% body weight

21.3. Pharmacogenomic Sub-study Protocol

21.3.1. Introduction

This is an exploratory pharmacogenomic research sub-study sponsored by Vanda Pharmaceuticals Inc. This sub-study is to be conducted according to United States and International Standards of Good Clinical Practice (FDA Title 21 part 312 and International Conference on Harmonization guidelines), applicable government regulations, and Institutional research policies and procedures.

Individuals respond differently to medications in terms of efficacy and adverse events. Differential drug response between individuals can be a consequence of genetic variation in genes involved in the metabolism or mechanism of action of the drug.

The pharmacogenomic whole genome scan addresses inter-individual variability in drug response by systematically correlating genetic variations (polymorphisms) spaced throughout DNA to an individual's response to a drug. The advantage of using the genome-wide pharmacogenomics approach is that no hypothesis is needed to choose the polymorphisms that will be typed in the sub-study.

The use of pharmacogenomics could eventually replace the trial-and-error strategy that is currently used in developing therapeutic strategies, by determining a priori who will respond favorably (or unfavorably) to a given type of drug treatment. This approach will ultimately lead to individualized medicine, in which drug treatment is tailored to suit an individual's genetic make-up.

The goal of this exploratory pharmacogenomic research sub-study is to identify genetic markers that may predict individuals with sleep disorders who will have the best response to VEC-162 and the least adverse events to maximize the benefit of VEC-162. The information generated from this sub-study will be used solely for research purposes to help develop safer and more effective treatment for sleep disorders. It will not be used to change diagnoses or alter therapy.

21.3.2. Objectives

Primary Objectives

To perform exploratory pharmacogenomics analysis on samples collected from patients consenting to this sub-study. Samples may be used to:

1. Identify genetic markers that correlate with response to VEC-162 treatment.
2. Identify genetic markers that correlate with adverse events that may occur upon treatment with VEC-162.

21.3.3. Study Design

To achieve the objectives described above, polymorphisms spaced throughout the genome will be correlated with relevant clinical parameters and clinically documented variations in drug response (efficacy and/or adverse events).

The sample may be stored for up to 15 years, under the control of the sponsor, for research on VEC-162, sleep disorders, and CNS only.

21.3.4. Patient Selection and Withdrawal

All patients enrolled in the VP-VEC-162-3104 research study will be given the option to participate in the Pharmacogenomic Sub-study. A patient may withdraw from the sub-study at any time by contacting his/her study Investigator, who will contact the sponsor. The sponsor or designee will be responsible for destroying any remaining sample materials and will send a letter back to the Investigator to confirm sample destruction. Any data or analysis generated from the sample prior to the request for destruction will not be destroyed. However, no new information will be generated from the sample and no new analysis will be performed.

21.3.5. Study Procedures

A blood sample will be drawn at Night 1 from each patient who agrees to participate in this sub-study to obtain sufficient DNA for the pharmacogenomic assessment. The blood is drawn into an EDTA tube(s) which will be gently inverted several times to prevent clotting. The sample(s) will then be placed in a -70°C (or -20°C) freezer at the site. The sample(s) will be shipped on dry ice to a central laboratory for DNA extraction. One aliquot of the DNA sample will be sent to Vanda for analysis. The sample will remain under the control of the sponsor. If the blood draw at Night 1 is missed, the sample can be collected at any other visit where a blood sample is already scheduled. The patient may be asked to supply an additional blood sample only if the original blood sample is lost or damaged.

21.3.6. Statistical Analysis

Correlation studies will be performed between parameters collected in the clinical database and genotypes for polymorphisms. Statistical tests to be performed may include Fisher's exact test, analysis of variance (ANOVA), and analysis of covariance (ANCOVA) using the same statistical models utilized for the primary and secondary objectives of the parent study, Protocol VP-VEC-162-3104. For significant associations, odds ratios and 95% confidence intervals will be calculated. Corrections for multiple testing will be made using appropriate methods that may include Bonferroni or bootstrapping. All statistical calculations will be carried out in the statistical program SAS (Cary, NC).

21.3.7. Data Handling and Confidentiality

Samples collected for pharmacogenomic studies will be double-coded by the following process: (1) The sample is collected at the doctor's office in a tube labeled with a barcode

- code #1. (2) The sample is then sent to a central lab for DNA extraction. An aliquot of the DNA sample is sent to Vanda where it will be identified with a new unique code – code #2. The sample is now double-coded. This maximizes patient confidentiality while allowing the patient the right to withdraw from the sub-study if they choose.

The DNA sample and any information generated from this sample will be labeled with the second code and no personal identifiers. In addition, all genetic information will be stored in a separate secured database, independent from the clinical data database. Only authorized personnel from Vanda will have access to the genetic data. In addition, the FDA or other regulatory agencies may request copies of the genetic data for auditing purposes. Unless ordered by a court of law, only the above mentioned agencies and personnel will have access to the genetic data.

Information about sub-study patients will be kept confidential and managed according to the requirements of the Health Insurance Portability and Accountability Act (HIPAA) of 1996. Information obtained from the sample will not be given back to the patient and will not be placed on the patient's medical record.

21.3.8. Ethical Considerations

All patients enrolled in the VP-VEC-162-3104 research study who agree to participate in the pharmacogenomic sub-study will be asked to sign a separate Pharmacogenomic Informed Consent. Choosing to not participate in this sub-study will not affect the patient's ability to participate in the main research study. The Pharmacogenomic Informed Consent will be submitted along with the main research study informed consent for review by the Institutional Review Board/Ethical Committee.

Vanda will obtain approval from the IRB/EC at each participating institution if they would like to perform additional research studies that are not outlined in this document.

21.3.9. Publication Plan

Any significant findings, based upon the analysis of aggregate data collected from this pharmacogenomic sub-study, may be published by Vanda Pharmaceuticals Inc. Patients will not be identified by any personal identifier in any publication resulting from this sub-study.
