# Supplementary material for: Melatonin agonist tasimelteon (HETLIOZ®) improves sleep in patients with primary insomnia: A multicenter, randomized, double-blind, placebo-controlled trial
Source: PLoS One. 2025 Sep 19;20(9):e0332366. doi: 10.1371/journal.pone.0332366 (PMC12449008; doi:10.1371/journal.pone.0332366)
Supplement: S1 Table — (PDF) [file pone.0332366.s003.pdf]

Supplemental Table 1: Summary of Rebound and Withdrawal Effects

Parameter	Placebo N=104		Tasimelteon 20 mg N=108		Tasimelteon 50 mg N=109	
**Latency to Persistent Sleep[a]**						
Estimate Mean Change (SEM)	-44.265 (4.962)*		-31.345 (4.817)*		-31.283 (4.927)*	
p-value (versus placebo)			0.060		0.062	
**Wake After Sleep Onset through Hour 6[a]**						
Estimate Mean Change (SEM)	-30.125 (4.543)*		-18.862 (4.444)*		-18.525 (4.565)*	
p-value (versus placebo)			0.074		0.070	
**Wake After Sleep Onset[a]**						
Estimate Mean Change (SEM)	-26.124 (5.507)*		-12.010 (5.346)*		-9.440 (5.497)	
p-value (versus placebo)			0.064		0.031	
**Total Sleep Time[a]**						
Estimate Mean Change (SEM)	60.325 (6.341)*		39.146 (6.153)*		38.412 (6.328)*	
p-value (versus placebo)			0.016		0.014	
**Sleep Efficiency[a]**						
Estimate Mean Change (SEM)	12.566 (1.317)*		8.166 (1.278)*		7.908 (1.315)*	
p-value (versus placebo)			0.016		0.012	
**Benzodiazepine Withdrawal Symptoms Questionnaire**						
	**Day 30**	**Day 37**	**Day 30**	**Day 37**	**Day 30**	**Day 37**
n	97	95	94	100	99	96
Mean (SD)	1.2 (2.1)	0.5 (1.3)	1.0 (2.0)	0.9 (2.6)	1.6 (3.4)	0.8 (1.5)
Median (range)	0 (0-8)	0 (0-6)	0 (0-10)	0 (0-15)	0 (0-18)	0 (0-7)

[a]Change from Baseline to Night 36 - ANCOVA Analysis (Modified Intent-to-Treat Population).Abbreviations: SD=Standard Deviation; SEM=Standard Error of the Mean; N = number in population; n = observed number.*Significant (p<0.05) change between baselines and night 36.
