# Supplementary material for: Melatonin agonist tasimelteon (HETLIOZ®) improves sleep in patients with primary insomnia: A multicenter, randomized, double-blind, placebo-controlled trial
Source: PLoS One. 2025 Sep 19;20(9):e0332366. doi: 10.1371/journal.pone.0332366 (PMC12449008; doi:10.1371/journal.pone.0332366)
Supplement: S2 Table — (PDF) [file pone.0332366.s004.pdf]

Supplemental Table 2: Summary of Next-Day Residual Effects.

Parameter	Placebo N=104	Tasimelteon 20 mg N=108	Tasimelteon 50 mg N=109
**Change from Baseline to Day 2**			
DSST	2.7 (7.4)	1.4 (8.6)	1.9 (9.6)
VAS Sad/Happy Domain	1.8 (12.1)	1.3 (13.0)	1.8 (12.4)
VAS Sleepy/Alert Domain	6.0 (17.4)	3.9 (19.9)	7.8 (19.8)
VAS Calm/Excited Domain	1.3 (13.9)	1.5 (13.3)	-0.2 (15.7)
**Change from Baseline to Day 9**			
DSST	2.6 (7.1)	1.7 (9.8)	2.2 (9.8)
VAS Sad/Happy Domain	1.8 (12.1)	1.3 (13.0)	1.8 (12.4)
VAS Sleepy/Alert Domain	6.7 (20.5)	3.6 (21.9)	7.3 (21.0)
VAS Calm/Excited Domain	2.3 (16.3)	1.3 (15.1)	-1.9 (18.5)
**Change from Baseline to Day 23**			
DSST	2.9 (8.2)	2.0 (8.1)	3.3 (11.7)
VAS Sad/Happy Domain	3.2 (13.8)	0.7 (15.4)	2.4 (16.6)
VAS Sleepy/Alert Domain	8.6 (18.4)	5.7 (23.3)	7.0 (21.9)
VAS Calm/Excited Domain	3.4 (15.8)	1.7 (16.8)	4.7 (20.5)
**Change from Baseline to Day 30**			
DSST	1.9 (8.5)	2.1 (9.3)	3.6 (9.5)
VAS Sad/Happy Domain	2.9 (14.2)	3.4 (15.0)	4.0 (16.5)
VAS Sleepy/Alert Domain	12.8 (19.6)	7.8 (23.4)	8.5 (24.9)
VAS Calm/Excited Domain	4.8 (17.6)	1.0 (18.0)	3.8 (22.3)

Abbreviations: DSST = Digit Symbol Substitution Test, VAS = Visual Analog Test *Note:* ANCOVA Analysis (Modified Intent-to-Treat Population) presented at Mean Change (SD). * p-value < 0.05; ** p-value < 0.01 (2-tailed).
