# Supplementary material for: Melatonin agonist tasimelteon (HETLIOZ®) improves sleep in patients with primary insomnia: A multicenter, randomized, double-blind, placebo-controlled trial
Source: PLoS One. 2025 Sep 19;20(9):e0332366. doi: 10.1371/journal.pone.0332366 (PMC12449008; doi:10.1371/journal.pone.0332366)
Supplement: S3 Table — (PDF) [file pone.0332366.s005.pdf]

Supplemental Table 3: Summary of Treatment-Emergent Adverse Events.

	Placebo N=104	Tasimelteon 20 mg N=109	Tasimelteon 50 mg N=109	Total N=322
Patients with at ≥1 TEAE	32 (30.8%)	39 (35.8%)	36 (33.0%)	107 (33.2%)
Patients with at ≥1 severe TEAE	3 (2.9%)	4 (3.7%)	3 (2.8%)	10 (3.1%)
Patients with at ≥1 suspected TEAE	13 (12.5%)	18 (16.5%)	18 (16.5%)	49 (15.2%)
Patients with ≥1 TEAE leading to study drug interruption	2 (1.9%)	1 (0.9%)	1 (0.9%)	4 (1.2%)
Patients with ≥1 TEAE resulting in study drug discontinuation	2 (1.9%)	3 (2.8%)	2 (1.8%)	7 (2.2%)
Patients with ≥1 TEAE resulting in study termination	2 (1.9%)	2 (1.8%)	2 (1.8%)	6 (1.9%)
Total number of SAEs	1	1	0	2
Patients with ≥1 SAE	1 (1.0%)	1 (0.9%)	0	2 (0.6%)

Data presented as (n (%)). Abbreviations: N = number in population; n = observed number; SAE = serious adverse events; TEAE = treatment-emergent adverse event.
