# Supplementary material for: Melatonin agonist tasimelteon (HETLIOZ®) improves sleep in patients with primary insomnia: A multicenter, randomized, double-blind, placebo-controlled trial
Source: PLoS One. 2025 Sep 19;20(9):e0332366. doi: 10.1371/journal.pone.0332366 (PMC12449008; doi:10.1371/journal.pone.0332366)
Supplement: Attachment — Submitted filename: Response to Reviewers.pdf [file pone.0332366.s008.pdf]

REVIEWER #1:

1. Reference
   14: JPharmSci Full name to be given,
   44: JAMA, Full name to be given,

Vanda Response

No. 14 has been updated to Journal of Pharmaceutical Sciences and no. 44 has been updated to Journal of the American Medical Association.

2. For Pairwise Chi-square contrast etc, the word contrast is to be replaced with another word.

Vanda Response

We have updated the text to: *'p-value (two-sided) from Pairwise Chi-square comparison of each active group'*.

3. The sentence 'The product of the adjusted baseline score by time as a covariate' requires revision e.g. an interaction term between the adjusted baseline score and time was included as a covariate

Vanda Response

We have updated the text to the suggested revision.

4. The sentence The treatment-by-baseline interaction' could be rephrased as 'interaction between treatment and baseline.' This suggestion applies throughout the manuscript

Vanda Response

We have updated the text to the suggested revision.
